# The application of *Bacillus amyloliquefaciens* and Arbuscular Mycorrhizal Fungi displays curative effects on Citrus Huanglongbing

**DOI:** 10.3389/fpls.2025.1636064

**Published:** 2025-08-29

**Authors:** Yuning Li, Yigang Lin, Zehua Liu, Maosheng Zeng, Hanhong Xu

**Affiliations:** National Key Laboratory of Green Pesticide/Key Laboratory of Natural Pesticide and Chemical Biology, Ministry of Education College of Plant Protection, South China Agricultural University, Guangzhou, China

**Keywords:** Citrus HuangLongBing, *Bacillus amyloliquefaciens*, Arbuscular Mycorrhizal Fungi, double inoculation, plant-pathogen interactions

## Abstract

Huanglongbing (HLB) is a severe citrus disease that results in significant yield loss, caused by the phloem-dwelling pathogen *Candidatus* Liberibacter asiaticus (*C*Las). Currently, antibiotics including penicillin, chloromycetin, and streptomycin are extensively utilized for the control of HLB in citrus production. However, various issues have emerged following the application of antibiotics, including the development of resistant microorganisms in soil and the accumulation of antibiotic residues in fruits. Consequently, there is an urgent need for alternative environmentally friendly methods for HLB control. In this study, the curative effects of *Bacillus amyloliquefaciens* strain HN11 and Arbuscular Mycorrhizal Fungi (AMF) on HLB were investigated. The HN11 strain and AMF demonstrated stable colonization within the interior of citrus roots. Moreover, HN11+AMF inoculation promoted the growth of HLB-infected citrus trees, accompanied by the decrease in *C*Las titers, reduction in H_2_O_2_ content, callus deposition, starch content, and ion leakage, as well as improvements in antioxidant enzyme activity indicators. The curative effects of HN11+AMF inoculation on HLB were also confirmed through field efficacy experiments. Moreover, HN11+AMF inoculation was identified to modify the composition and functions of root-soil bacterial and fungal microbial communities. These findings indicate that the use of HN11+AMF inoculation may serve as a promising control strategy for HLB management.

## 1 Introduction

Huanglongbing (HLB) is a destructive bacterial disease that significantly affects global citrus production, leading to substantial ecological and economic losses (Gottwald et al., 2012; Alquézar et al., 2022). The disease is attributed to the pathogen *Candidatus* Liberibacter spp. and is transmitted between trees by two vectors: the Asian citrus psyllid (ACP) *Diaphorina citri* Kuwayama (Hemiptera: Psyllidae) and the African citrus psyllid (AfCP) *Trioza erytreae* Del Guercio (Hemiptera: Triozidae) (Pérez-Hedo et al., 2025). The pathogen, upon colonizing the phloem tissue, disrupts the function of sieve elements, restricts carbohydrate transport, and leads to root deterioration, ultimately causing plant stunting and dieback (Li et al., 2006; Achor et al., 2010; Zhou, 2020). HLB impacts most commercial citrus cultivars, presenting symptoms such as uneven pigmentation, reduced flavor, heightened acidity in the fruit, root degeneration, and leaf chlorosis (Paula et al., 2018; Dala-Paula et al., 2019; Laranjeira et al., 2020). Since its discovery in the 1980s, HLB has consistently threatened China. It has spread across multiple provinces, resulting in significant destruction of citrus orchards (Graham et al., 2013). Approximately 50 million infected trees have been eliminated to date, significantly hindering the advancement of China’s citrus industry (Zhou, 2020).

HLB control depends on integrated approaches because there are no resistant citrus cultivars or effective curative medicines. Lin Kongxiang initially introduced the “Three-Step” technique, which is still a mainstay of HLB control and emphasizes the creation of nurseries free of disease, the removal of diseased trees, and strict psyllid population control. To lessen the sickness, other tactics have been investigated, including the use of antibiotics, heat therapy, transgenic techniques, and dietary management. Although antibiotic treatments, such as sulfonamides and penicillin derivatives, have demonstrated promise in lowering bacterial titers in infected trees, their widespread use is constrained by regulatory limits, environmental effects, and worries about antibiotic residues (Hussain et al., 2019; Yang et al., 2020). Although studies have shown that exposing infected plants to 48–51°C can temporarily reduce bacterial loads, field-scale implementation is still difficult due to inconsistent temperature control and logistical challenges. Heat treatment, which takes advantage of the pathogen’s thermal sensitivity, has been tested under controlled conditions. The insertion of resistance genes by Agrobacterium-mediated transformation has been investigated in genetic engineering efforts; some encouraging findings indicate that transgenic citrus plants have lower bacterial burdens (Hao et al., 2016). However, widespread use of genetically modified crops is hampered by their high costs and regulatory obstacles. Enhancing plant defense responses against HLB has been suggested through nutrient supplementation, especially with elements like zinc and manganese. However, this strategy is not a long-term solution because the pathogen persists and spreads if infected trees and psyllid populations are not adequately managed. Recently, the therapeutic small peptides with curative effects on HLB were mined (Zhao et al., 2025). While the stability and safety of these peptides need to be further studied.

Due to the constraints of above control methods, there is increasing interest in environmentally sustainable alternatives, especially microbial biocontrol. Beneficial microorganisms, such as plant growth-promoting rhizobacteria (PGPR) and Arbuscular Mycorrhizal Fungi (AMF), have demonstrated potential in alleviating HLB by bolstering plant immunity, competing with the pathogen, and strengthening soil health (Niu et al., 2020; Munir et al., 2022a). *Bacillus* species, recognized a class of PGPRs with the potent biocontrol capabilities, are extensively utilized in disease suppression owing to their capacity to generate antimicrobial compounds, stimulate systemic resistance, and enhance plant growth. Many species have been identified to be effective for HLB control. For example, inoculation with *Bacillus* spp., specifically *B. subtilis* L1-21, has shown efficacy in diminishing bacterial titers in infected citrus trees (Munir et al., 2022b). In addition, *B. amyloliquefaciens* GJ1 exhibits the antibacterial potential against citrus HLB, which could be related to improving the immunity of citrus by increasing the photosynthesis and enhancing the expression of the resistance-related genes (Nan et al., 2021). AMF, which form symbiotic associations with plant roots, have been documented to augment nutrient absorption, bolster stress resilience, and influence microbial populations in the rhizosphere, hence enhancing disease resistance (Tabin et al., 2009; Weng et al., 2022). It was reported that citrus inoculation with AMF alleviate the stress induced by HLB, including reduction in the content of *C*Las and malondialdehyde (MDA) in the leaves, significantly enhanced peroxide (POD) activity in the leaves, and increased the proline (Pro) content, superoxide dismutase (SOD) activity, and catalase (CAT) content in both the leaves and roots (Tang et al., 2023). However, the effects of *Bacillus* species and AMF application on HLB remain unknown.

In this study, our aim was to investigate the curative effects of *B. amyloliquefaciens* strain HN11 and AMF on HLB. At first, the pot experiment was performed to determine the colonization effect of this strain within the interior of citrus roots was analyzed. The physiological parameters of HLB-infected trees altered by HN11 and AMF application were also determined, including *C*Las titers, H_2_O_2_ content, callus deposition, starch content, ion leakage, antioxidant enzyme activities. Besides, the curative effects of HN11 and AMF on HLB-infected trees were further investigated in field efficacy experiments. Furthermore, the changes of root-soil microbial communities induced by HN11 and AMF application were also analyzed. Our results suggest that the co-inoculation of HN11 and AMF represents a promising approach for HLB management.

## 2 Materials and methods

### 2.1 Bacterial strain and culture conditions

*B. amyloliquefaciens* HN11 was extracted from the roots of the medicinal plant *Azadirachta indica* and archived at the China General Microbiological Culture Collection Center in Beijing (Accession number: CGMCC15726). Its morphological characteristics are detailed in **Supplementary Figure 1**. *B. amyloliquefaciens* HN11 and its GFP-tagged variant were preserved in 40% glycerol (v/v) at −80°C; the stock culture was replenished every four months on Luria Bertani (LB) agar and incubated for 24–48 hours to assess the stability of HN11. A pure culture of HN11 was cultivated in LB broth for 12 hours at 37°C in a shaking incubator at 200 rpm until the late logarithmic phase was reached. Inoculum of AMF was procured from BioOrganics™ (New Hope, PA, USA). It comprised a mixture of rhizophagus intraradices. The inoculum was assured to contain at least 30 spores per milliliter.

### 2.2 Greenhouse pot experiment

Four categories of agents were utilized: (1) sterile-CK, (2) *B. amyloliquefaciens* inoculation-HN11, (3) arbuscular mycorrhizal fungi inoculation-AMF, and (4) *B. amyloliquefaciens* combined with AMF inoculation-HN11+AMF. One-year-old citrus trees of the fertile orange variety were utilized for the experiment. The trees exhibited an average height of 52 cm and an average diameter of 8 mm. Citrus potted plants were inoculated with HLB by grafting techniques and sustained under infection for more than three months. Bacteriological research commenced following the confirmation of HLB-positive status in seedlings via standardized detection procedures. No significant difference in the *C*Las titers of the trees used in this experiment. For the HN11+AMF group, the dosage of HN11 used in this experiment is 10^8^ CFUeri^-1^ per plant, while the water consumption is 400 mL per plant with simulated drip irrigation technology utilized. 100 g of AMF is administered per citrus tree, uniformly distributed over the rhizosphere via a hole application approach. Both microbiological agents were administered on April 1, 2021. The HN11 bacterial agent was administered biweekly for a total of four applications; the AMF microbial agent was applied monthly, treated with two times overall. For the CK group, equal amount of water was dripped per plant. For the HN11 group, only equal amount of HN11 was applied, and only equal amount of water and AMF was applied in the AMF group. Six citrus trees were processed as one replicate, and three biological replicates performed for each group. The matrix soil was utilized in the experiment, and the ambient condition is a constant temperature of 28°C within a greenhouse. Strict water and fertilizer and pest management during the experiment, and no *D. citri* was observed on the experimental materials.

#### 2.2.1 Sample collection and microbial colonization analysis

Following 60 days of HN11 and AMF treatment on potted citrus plants, samples of leaves and roots were collected for the assessment of microbial colonization.

To conduct a preliminary investigation of the control mechanism of HN11 against Huanglongbing, diseased citrus leaves and root tissues were treated with the HN11-GFP strain as described in section 2.1, and the treated leaves and root tissues were examined using a laser confocal microscope. Subsequent to the fungal treatment, the samples were meticulously cleaned with water, and the midribs and roots of the citrus leaves were subjected to embedding solution and sectioned using a cryostat. The samples were examined with a laser confocal microscope at excitation/emission wavelengths of 488 nm/500–550 nm, and the procedure was replicated three times.

The experiment on bacterial rhizosphere colonization is cited from Tian et al (Zhou, 2020). Administer 400 mL of freshly cultivated HN11 diluted bacterial solution (1.0×10^8^ CFU mL^-1^) to citrus potted plants biweekly for a total of four applications. After 60 days, the citrus roots were collected and cleansed with sterile water. About 1 cm of root samples from each treatment was preserved in sterile Eppendorf tubes. Cryo-scanning electron microscopy and plate recovery enumeration were used for the observation of HN11. In addition, the root samples of citrus potted plants subjected to microbial agents for a duration of 60 days were also gathered, then the trypan blue staining technique was employed to assess the AMF infection rate in citrus roots (Lee et al., 2023).

#### 2.2.2 Detection of *C*Las titers by fluorescence quantitative PCR

After 60 days of different treatments, the DNA of the root samples was isolated with the OMEGA D2485–04 Plant DNA Small Volume Extraction Kit. Fluorescence quantitative PCR detection was conducted utilizing the primers *C*Las-4G/HLBr and the HLB Probe as reported by Bao et al (Wang et al., 2022). The Ct values derived by amplifying the recombinant plasmid with a 10-fold gradient dilution as a template served as the standard curve to quantify the *C*Las copy number in each sample. The concentration of *C*Las in each sample is quantified as the copy number per nanogram of total DNA. The quantity of *C*Las copies per gram of sick citrus leaf material was determined using a standard curve value.

#### 2.2.3 Plant growth index monitoring

The height, stem diameter, root length, dry mass, fresh mass, and leaf area of citrus trees were quantified in different treatment groups. The root-shoot ratio and biofeedback were assessed using the methodology outlined by Wang et al (Adhikary et al., 2019). The chlorophyll concentration was assessed by measuring absorbance at 645 and 663 nm, following the Adhikary method (Zhao et al., 2010). The citrus roots were examined using a root scanner and assessed using WinRHIZO root analysis software. Root activity was assessed utilizing the TTC reduction technique (Ma et al., 2022). A soil pH meter is utilized to assess soil acidity levels.

#### 2.2.4 Plant defense response analyses

The assessment of reactive oxygen species (ROS) and callose deposition in different treatment groups was performed as described by Ma et al (Bao et al., 2019). Hydrogen peroxide (H_2_O_2_), superoxide dismutase (SOD), peroxidase (POD), catalase (CAT), and glutathione (GSH) were quantified utilizing the micro H_2_O_2_ test kit, SOD assay kit, POD assay kit, CAT assay kit, and GSH assay kit supplied by Beijing Solarbio Science & Technology Co., Ltd.

### 2.3 Field experiments

Field experiments were performed in citrus orchards located at 23.169°N, 114.996°E in Huizhou City, Guangdong Province. Four categories of microbial agents were utilized: (1) sterile-CK, (2) *B. amyloliquefaciens* inoculation-HN11, (3) arbuscular mycorrhizal fungi inoculation-AMF, and (4) *B. amyloliquefaciens* combined with arbuscular mycorrhizal fungus inoculation-HN11+AMF. Prior to administering the microbial agent, identify infected plants exhibiting generally uniform levels of HLB, assign labels, and assess the incidence among the plants. The citrus plant is 4 years old, with an average height of 221.99 cm and an average crown width of 407.88 cm. The prevalence of citrus HLB in the field exceeds 80%. In this study, the trees with similar *C*Las titers were selected for subsequent experiments. The dosage of *B. amyloliquefaciens* HN11 is 10^8^ CFU mL^-1^ per plant, and the water requirement is 10 L per plant. It is administered via drip irrigation apparatus. The dosage of AMF is 1000 g per plant, administered uniformly around the rhizosphere utilizing a hole application technique. Both agents were administered on December 7, 2021. The HN11 bacterial agent was administered biweekly, with a total of 15 times conducted from December 2021 to July 2022. The AMF bacterial agent treatment was administered bi-monthly, with a total of three times conducted from December 2021 to July 2022. 10 citrus trees were used as one replicate for each treatment group, with three independent replicates. Strict water and fertilizer and pest management during the experiment to control the infestation or infection via *D. citri*. The *C*Las titers, citrus tree vigor, and condition in different treatment groups were determined and recorded every 2 months. The methods for *C*Las titers determination was according to part 2.2.2.

### 2.4 Analysis of soil microbial community

#### 2.4.1 Experimental procedure

After six-month treatments, the rhizosphere soil about 10 cm underground were collected for 16S and ITS high-throughput sequencing. The sequencing was conducted by the commercial service provider, Guangzhou Gene Denovo Biotechnology Co., Ltd.

Total DNA in the collected soil samples was extracted according to the instructions provided by the soil DNA extraction kit. The precise steps are as follows: 0.25–0.5 g of soil sample and 0.6 mL of Buffer SOL were added into a 2 mL bead tube, and vortex at peak velocity for 5 to 10 min. 60 µL of Buffer SDS was then added into the sample and vortexed for 15 s. After incubating at 70°C for 10 min, 200 µL of Buffer PS and 150 µL of Absorber Solution were added into the tube. Vortex for 20 s and incubation at room temperature for 5 min. After centrifugation at 13,000 × g for 5 min, the supernatant was transferred to a 2 mL centrifuge tube. An equivalent volume of Buffer GDP was added and mix by inversion. The DNA was then filtered using a collection column by centrifuging at 13,000 × g for 60 s, washed with Buffer GDP and Buffer GW2 and centrifuged at 13,000 × g for 60 s. Finally, 50 µL of Buffer AE, preheated to 70°C, was used to dissolve the DNA. The DNA was stored at 2–8°C for short-term utilization or at −20°C for extended storage. The agarose gel electrophoresis was used to evaluate the quality of the extracted DNA. The DNA purity and concentration was determined via a NanoDrop spectrophotometer.

Diluted genomic DNA was utilized to amplify the soil bacterial 16S rDNA and soil fungus ITS sections using appropriate primers. The primer sequences are enumerated in **Table 1**. The PCR reaction technique for amplifying 16S rRNA and ITS was fundamentally the same, differing only in the primers used, and is outlined as follows: 4 μL of 5× TransStart FastPfu Buffer, 2 μL of 2.5 mM dNTPs, 0.8 μL of 5 μM forward primer, 0.8 μL of 5 μM reverse primer, 0.4 μL of TransStart FastPfu DNA Polymerase, and 10 ng of genomic DNA. The PCR amplification method was conducted at 95°C for 3 min, followed by 27 cycles of 95°C for 30 s, 55°C for 30 s, and 72°C for 30 s. This was succeeded by a final extension at 72°C for 10 min, after which the samples were maintained at 4°C. Three technical replicates were conducted for each sample, and three biological replicates were executed for each treatment.

**Table 1:** The primers used in this study.

Type	Region	Primer name	Primer sequence (5’-3’)
*C*Las	16s rRNA	*C*Las 4G HLBr HLB-Probe	5’-AGTCGAGCGCGTATGCGAAT-3’ 5’-GCGTTATCCCGTAGAAAAAGGTAG-3’ 5’-FAM-AGACGGGTGAGTAACGCG-BHQ1-3’
16S	V3-V4	341F	5’-CCTACGGGNGGCWGCAG-3’
		806R	5’-GGACTACHVGGGTATCTAAT-3’
ITS	ITS2	ITS3_KYO2	5’-GATGAAGAACGYAGYRAA-3’
		ITS4	5’-TCCTCCGCTTATTGATATGC-3’

AMPure XP Beads were utilized to purify the amplified product, and the ABI Stepone Plus Real-time PCR equipment (Life Technologies, USA) was employed for quantification. The pure amplified product (amplicon) was ligated with a sequencing linker to create a sequencing library, and Illumina technology was employed for online sequencing.

#### 2.4.2 Information analysis

Following the sequencing of raw reads, the Usearch program was utilized for data processing. The low-quality reads or non-biological data were discarded first. Subsequently, the paired-end reads were concatenated into a tag, followed by the filtration of low-quality tags. The Usearch software was utilized to cluster and remove the chimeric tags detected throughout the clustering procedure to ascertain the abundance and representative sequence of the OTU. Species annotation, species composition analysis, indicator species analysis, alpha diversity analysis, beta diversity analysis, and community function prediction are performed utilizing the sequencing and abundance data of operational taxonomic units (OTUs). Valid groups are compared, and the variances among them are subjected to statistical examination.

### 2.5 Statistical analysis

Statistical analysis was performed using SPSS 26.00 software, and the data obtained for each group was expressed as mean ± SE. When the data conformed to normal distribution, the independent sample t-test was used for comparative analysis between two groups; one-way ANOVA was used for comparative analysis between multiple groups, and DMRT method was used for two-way comparison between groups. Otherwise, Mann–Whitney *U* test was used for comparison between two groups; Kruskal–Wallis *H* test was used for comparison between multiple groups. The test level was set at α = 0.05. When *p* < 0.05, the result statistics were significantly different.

## 3 Results

### 3.1 HN11 and AMF stable colonization within the interior of citrus roots and promoted the growth of HLB-infected citrus trees in the pot experiments

#### 3.1.1 HN11 and AMF stable colonization within the interior of citrus roots

**Figure 1:**
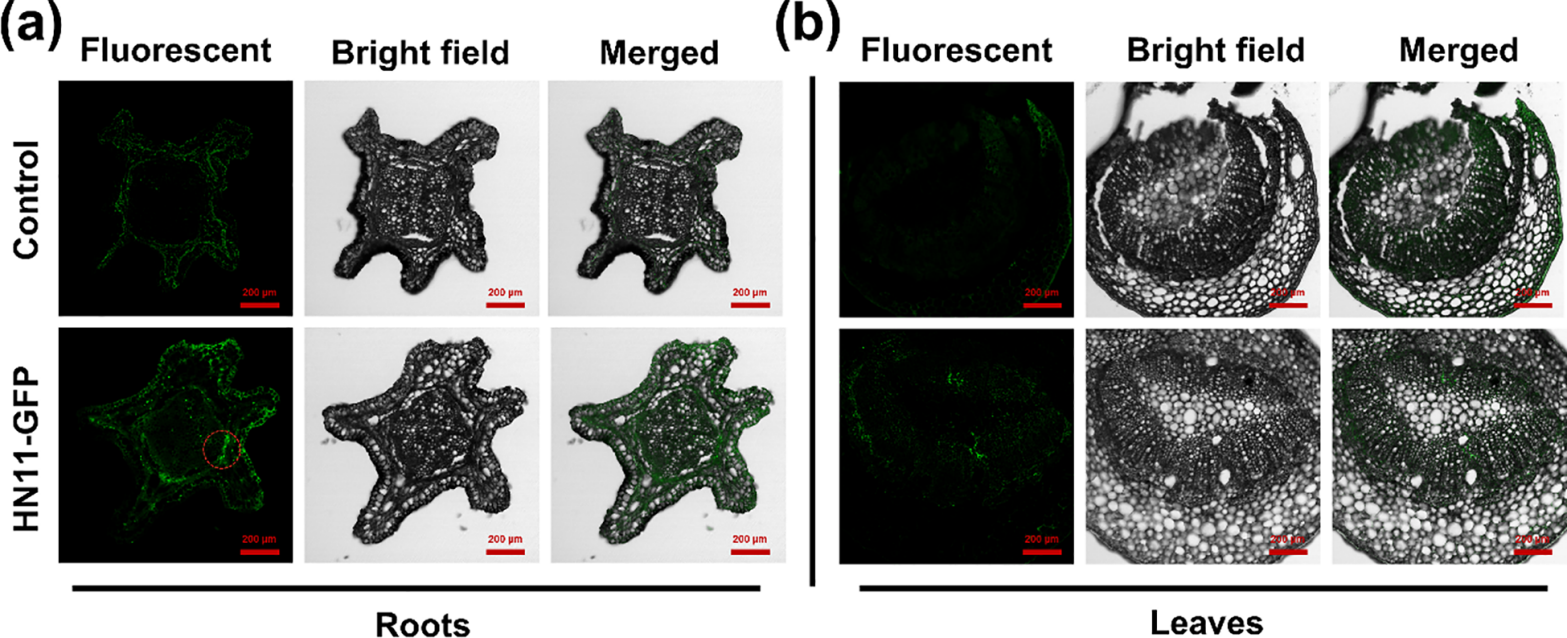
Confocal laser imaging. **(a)** Analysis of HN11-GFP colonization in citrus roots; **(b)** Analysis of HN11-GFP colonization in citrus leaf veins. Red circles indicate the colonization of GFP-tagged HN11 in the roots. HN11-GFP fluorescence is usually much stronger than plant autofluorescence, so it can be distinguished by comparing fluorescence intensity. Sample size n = 6 in the figure.

To initially elucidate the regulatory mechanism of *B. amyloliquefaciens* HN11 against *C*Las. The laser confocal microscopy was employed to examine the colonization of HN11-GFP in citrus roots and foliage. **Figure 1a** illustrates that five days post-inoculation of the bacterial agent, HN11-GFP strains were detected in the roots, whereas **Figure 1b** indicates the absence of HN11-GFP strains in the leaves. The findings indicated that HN11 had a greater propensity for colonization within the root system.

**Figure 2:**
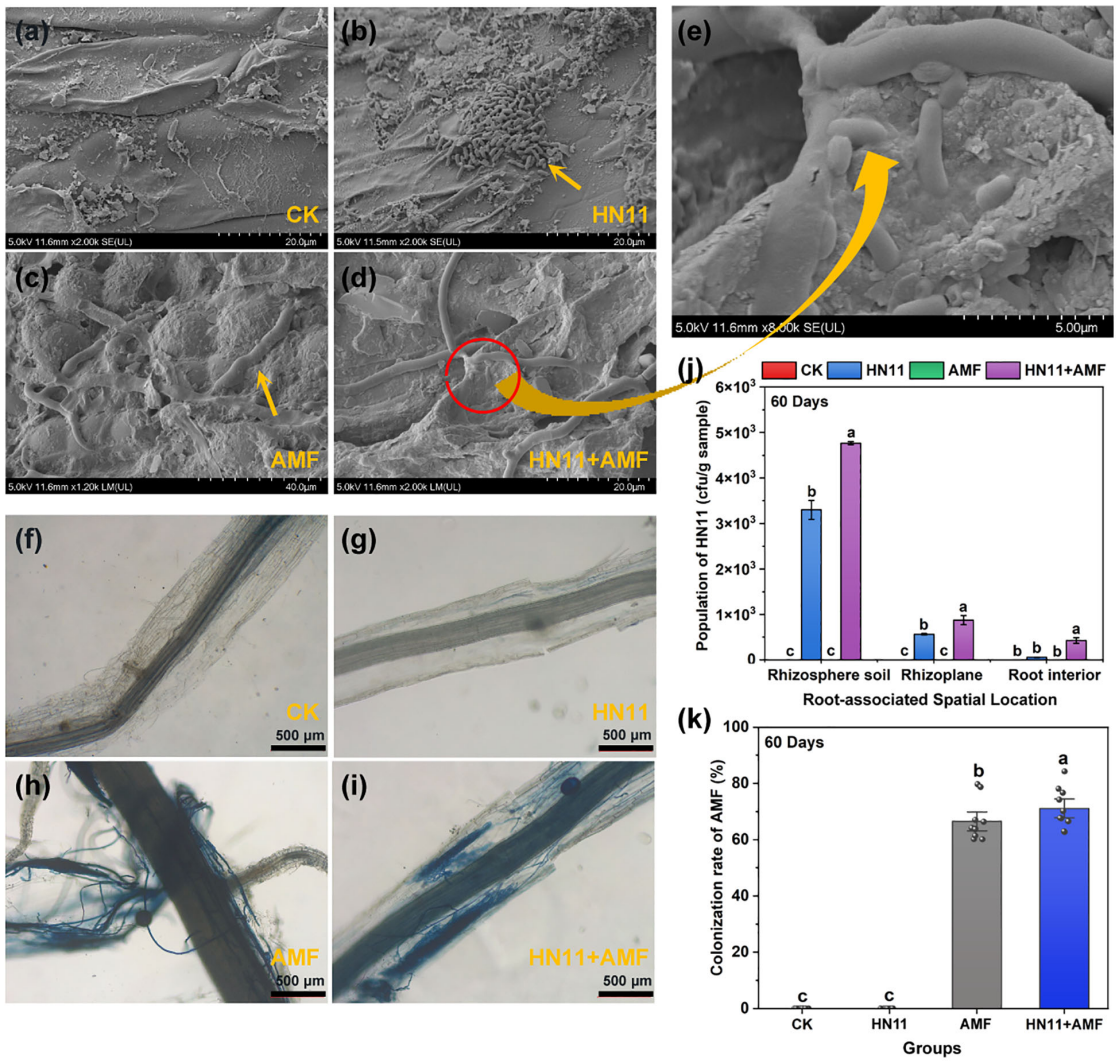
Colonization of HN11 and AMF in citrus roots **(a)** Cryo-EM image of CK treatment group **(b)** Cryo-EM image of HN11 treatment group **(c)** Cryo-EM image of AMF treatment group **(d)** Cryo-EM image of HN11+AMF treatment group **(e)** Cryo-EM magnified image of HN11+AMF treatment group **(f)** Optical microscope image of CK treatment group **(g)** Optical microscope image of HN11 treatment group **(h)** Optical microscope image of AMF treatment group **(i)** Optical microscope image of HN11+AMF treatment group **(j)** Colonization abundance of HN11 **(k)** Infection rate of AMF.CK is the control group, HN11 is the HN11 single inoculation treatment group, AMF is the AMF single inoculation treatment group, and HN11+AMF is the HN11+AMF double inoculation treatment group. The data in the figure are Mean ± SE, and one-way ANOVA was used for statistical analysis. Different letters indicate significant differences between different treatments (*p*<0.05). Sample size n = 10.

Cryo scanning electron microscopy revealed the absence of HN11 and AMF in the control group (**Figure 2a**); HN11 exhibited extensive colonization in the roots, forming microcolonies (**Figure 2b**); AMF effectively infected citrus roots (**Figure 2c**); concurrently, HN11 was found to accumulate at the junctions of AMF-infected roots (**Figures 2d, e**). HN11 was isolated using the plate dilution technique. The quantities of HN11 in the rhizosphere soil, on the root surface, and within the root of citrus subjected to single inoculation were 3.3×10³, 5.6×10², and 5.8×10^1^ CFU g^-^¹, respectively. The quantities of HN11 in the rhizosphere soil, on the root surface, and within the root of citrus subjected to double inoculation were 4.8×10³, 8.7×10², and 4.3×10² CFU g^-^¹, respectively. The population size was markedly greater than that of the single inoculation treatment (**Figure 2j**). In addition, the colonization of AMF in the roots with different treatments was also analyzed by trypan blue staining and observed using optical microscopy. No staining was observed in the roots in the CK group and HN11 treatment group, while significant blue staining was identified in the AMF and HN11+AMF treatment groups. Besides, a profusion of hyphae and branching structures in the citrus root system following treatment with AMF and HN11+AMF (**Figures 2f-i**). These results suggest that AMF colonized in the citrus roots after AMF and HN11+AMF treatments. Moreover, the infection rate of AMF in the HN11+AMF double inoculation therapy (71.10%) exceeded that of the AMF single inoculation (66.51%), suggesting that HN11 could enhance the colonization of AMF in the citrus roots (**Figure 2k**). These findings indicated that HN11 and AMF could effectively colonize the roots of citrus, with the dual inoculation of HN11 and AMF demonstrating superior efficacy compared to single inoculation treatments.

#### 3.1.2 HN11 and AMF application inhibited the *C*Las titers

This study performed a quantitative examination of *C*Las content following treatment with different agents, utilizing fluorescence quantitative PCR to assess their potential to mitigate symptoms of HLB in citrus. **Table 2** illustrates the alteration of *C*Las content within the veins. Between 15 and 60 days, the concentration of *C*Las in the veins exhibited an upward trajectory; however, the rate of increase in the HN11 and HN11+AMF inoculation treatments was slower compared to the control group and AMF injection. On the 60th day, the concentration of *C*Las in the veins of citrus for the CK, HN11, AMF, and HN11+AMF treatment groups was 1.67×10^5^, 3.74×10^4^, 8.15×10^4^, and 3.86×10^4^ copies/ng DNA, respectively. In comparison to the control group, the concentration of *C*Las in the HN11, AMF, and HN11+AMF treatments diminished by 6.3×10^4^, 1.9×10^4^, and 6.2×10^4^ copies/ng DNA, respectively. Between 15 and 60 days, the concentration of *C*Las in the root system exhibited an upward trajectory; however, the rate of increase in the HN11 and HN11+AMF inoculation treatments was slower compared to the control group and AMF inoculation treatments. On the 60th day, the concentrations of *C*Las in the roots of citrus for the CK, HN11, AMF, and HN11+AMF treatment groups were 4.68×10^5^, 5.53×10^4^, 9.23×10^4^, and 3.6×10^4^ copies/ng DNA, respectively. In comparison to the control group, the levels of *C*Las in the HN11, AMF, and HN11+AMF treatments decreased by 2.8×10^5^, 2.5×10^5^, and 3.0×10^5^ copies/ng, respectively. This suggests that the inoculation of agents can enhance the progression of HLB citrus root symptoms.

**Table 2:** Changes in *C*Las content (pot experiment).

Tissue	Time	Parameter	CK	HN11	AMF	HN11+AMF
Leaves	0 d	*C*Las	Nd	Nd	Nd	Nd
	15 d	*C*Las	6.09×10^3^ ± 2.34×10^3^ **^a^**	7.77×10^3^ ± 2.87×10^3^ **^a^**	8.11×10^3^ ± 2.22×10^3^ **^a^**	9.67×10^3^ ± 5.01×10^3^ **^a^**
	30 d	*C*Las	2.19×10^4^ ± 4.14×10^3^ **^a^**	1.60×10^4^ ± 1.09×10^3^ **^a^**	1.59×10^4^ ± 4.68×10^3^ **^a^**	1.31×10^4^ ± 5.18×10^3^ **^a^**
	60 d	*C*Las	1.67×10^5^ ± 1.41×10^4^ **^a^**	3.74×10^4^ ± 9.41×10^3^ **^b^**	8.15×10^4^ ± 1.87×10^4^ **^ab^**	3.86×10^4^ ± 2.35×10^4^ **^b^**
Roots	0 d	*C*Las	2.88×10^3^ ± 1.60×10^3^ **^a^**	4.30×10^3^ ± 1.30×10^3^ **^a^**	3.69×10^3^ ± 1.92×10^3^ **^a^**	4.04×10^3^ ± 1.14×10^3^ **^a^**
	15 d	*C*Las	3.80×10^3^ ± 7.13×10^2^ **^a^**	1.75×10^3^ ± 5.62×10^2^ **^a^**	2.63×10^3^ ± 6.78×10^2^ **^a^**	9.01×10^2^ ± 3.13×10^2^ **^a^**
	30 d	*C*Las	1.27×10^4^ ± 2.69×10^3^ **^a^**	3.88×10^3^ ± 8.71×10^2^ **^a^**	1.00×10^4^ ± 7.72×10^3^ **^a^**	2.15×10^3^ ± 8.37×10^2^ **^a^**
	60 d	*C*Las	4.68×10^5^ ± 1.07×10^5^ **^a^**	5.53×10^4^ ± 4.52×10^4^ **^b^**	9.23×10^4^ ± 6.51×10^4^ **^ab^**	3.59×10^4^ ± 1.72×10^4^ **^b^**

Different letters indicate differences between groups (p<0.05). The *C*Las unit is copies/ng DNA. The data presented in the table is Mean ± SE. The sample size in the figure is n=4. Nd, not detected. CK is the control group, HN11 is the HN11 single inoculation treatment group, AMF is the AMF single inoculation treatment group, and HN11+AMF is the HN11+AMF double inoculation treatment group.

#### 3.1.3 HN11 and AMF application enhanced the growth of HLB-infected citrus trees

**Figure 3:**
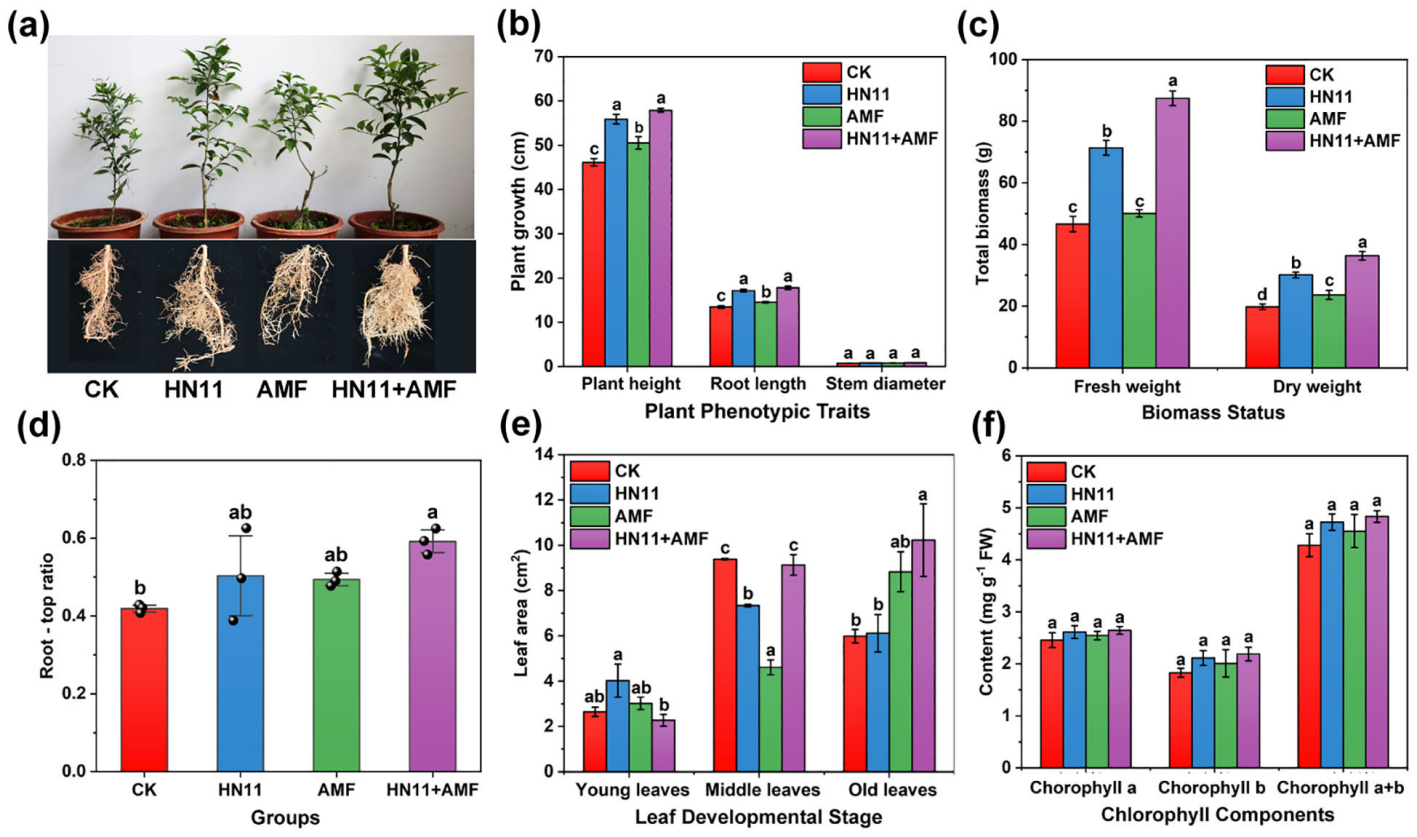
Growth indicators of potted citrus after bacterial treatment **(a)** Citrus growth **(b)** Plant height, stem diameter and root length **(c)** Fresh weight and dry weight **(d)** Root-crown ratio **(e)** Leaf area **(f)** Chlorophyll content. CK is the control group, HN11 is the HN11 single inoculation treatment group, AMF is the AMF single inoculation treatment group, and HN11+AMFis the HN11+AMF double inoculation treatment group. The data in the figure are Mean ± SE, and One-way ANOVA was used for statistical analysis. Different letters indicate significant differences between different treatments (*p*<0.05). Sample size n=3 in the figure.

As shown in **Figure 3a**, the afflicted citrus fruits following different microbial treatments exhibited enhanced growth compared to the control group (**Figure 3a**). In comparison to the control group, inoculation with HN11, AMF, and HN11+AMF resulted in increases in citrus plant height of 21.0%, 9.4%, and 25.4%, respectively, and root length increases of 27.5%, 8.0%, and 32.3%, respectively (**Figure 3b**). The fresh and dry weights of citrus fruits subjected to HN11+AMF double inoculation were markedly superior to those of the other treatment groups (**Figure 3c**). The root-to-shoot ratio of HN11+AMF inoculation group was the highest, demonstrating the benefits of the dual inoculation treatment (**Figure 3d**). In comparison to the control group, the HN11+AMF dual inoculation treatment enhanced the recovery of old citrus leaves (**Figure 3e**). Nonetheless, the HN11+AMF dual inoculation treatment did not significantly influence the levels of chlorophyll a, chlorophyll b, and total chlorophyll in the leaves (**Figure 3f**).

In comparison to the control group, the root activity and quantity of primary new roots in the HN11+AMF double inoculation achieved their peak values of 1136.59 μg g^-1^ h^-1^ and 135.67 strips, respectively. The overall root length of citrus roots augmented by 0.56 times, the total root surface area expanded by 1.04 times, the total number of root tips rose by 0.4 times, and the quantity of pointed tips climbed by 0.96 times when compared to the control group (**Table 3**). In addition, the efficacy of each treatment group in promoting root growth is ranked as follows: HN11+AMF > HN11 > AMF > CK.

**Table 3:** Root growth indicators of pot experiment.

Root growth indicators	CK	HN11	AMF	HN11+AMF
Root activity/μg g^-1^ h^-1^	310.06 ± 34.39 **^c^**	658.66 ± 37.89 **^b^**	390.57 ± 38.57 **^c^**	1136.59 ± 87.32 **^a^**
New roots/strip	52.67 ± 3.28 **^d^**	106.33 ± 9.6 **^b^**	81.33 ± 8.74 **^c^**	135.67 ± 5.81 **^a^**
Total root length/cm	441.44 ± 26.48 **^c^**	615.69 ± 33.92 **^ab^**	540.97 ± 21.35 **^b^**	689.09 ± 28.40 **^a^**
Total root surface area/cm^2^	99.89 ± 11.93 **^b^**	140.97 ± 11.34 **^b^**	126.06 ± 7.65 **^b^**	203.50 ± 14.02 **^a^**
Average root diameter/mm	0.83 ± 0.02 **^a^**	0.82 ± 0.02 **^a^**	0.88 ± 0.03 **^a^**	0.80 ± 0.03 **^a^**
Root tips/number	520.72 ± 22.34 **^c^**	643.00 ± 43.07 **^ab^**	547.47 ± 20.19 **^bc^**	729.09 ± 30.95 **^a^**
Root apex/number	246.34 ± 26.52 **^b^**	395.95 ± 26.09 **^a^**	284.13 ± 34.38 **^b^**	484.02 ± 18.32 **^a^**
Root crossing/number	19.27 ± 1.76 **^a^**	23.97 ± 4.50 **^a^**	21.66 ± 3.31 **^a^**	20.80 ± 3.12 **^a^**

Different letters indicate differences between groups (p<0.05). n=5. CK is the control group, HN11 is the HN11 single inoculation treatment group, AMF is the AMF single inoculation treatment group, and HN11+AMF is the HN11+AMF double inoculation treatment group.

#### 3.1.4 HN11 and AMF application altered the plant defense responses

**Figure 4:**
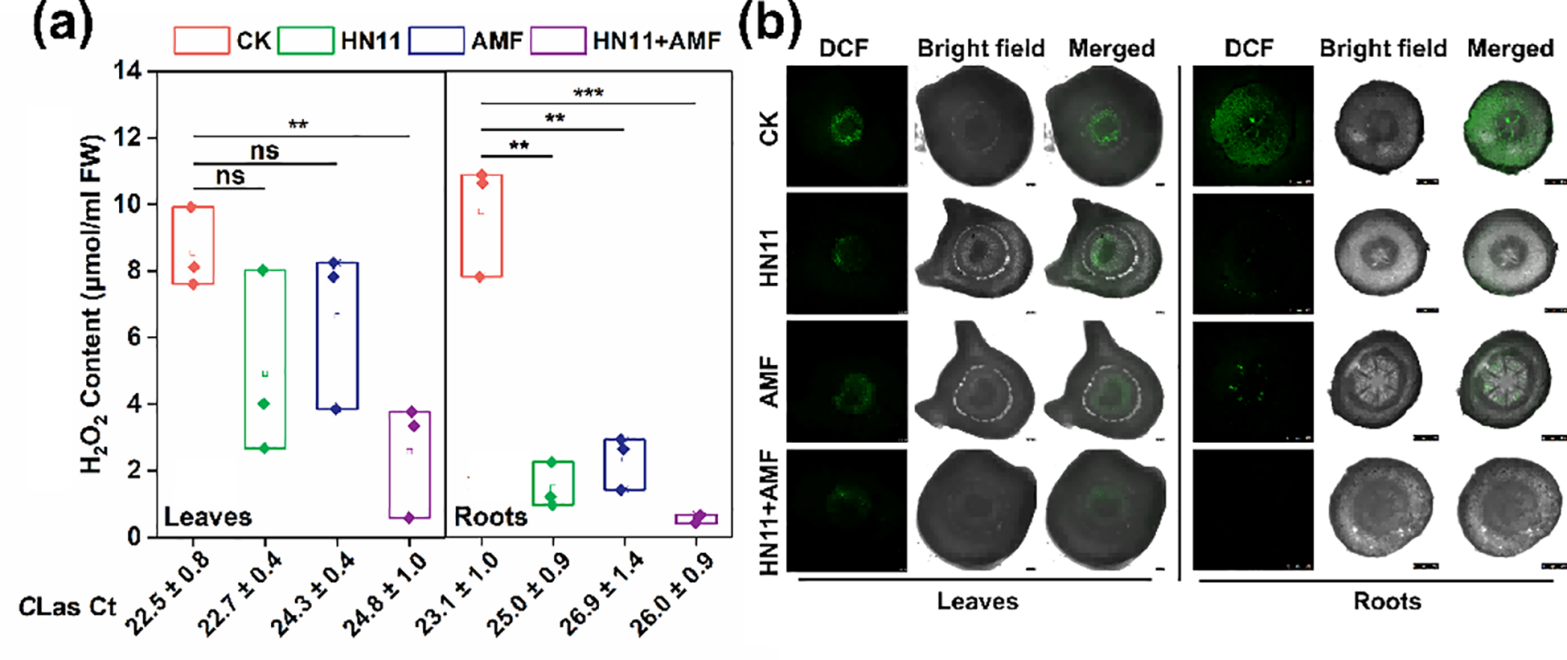
Hydrogen peroxide content and reactive oxygen species (ROS) localization in leaf and root tissues after bacterial treatment **(a)** Hydrogen peroxide content **(b)** Observation of reactive oxygen species (ROS) localization. The larger the Ct value, the lower the content of Huanglongbing bacteria. CK is the control group, HN11 is the HN11 single inoculation treatment group, AMF is the AMF single inoculation treatment group, and HN11+AMF is the HN11+AMF double inoculation treatment group. **, *** and ns indicate *p*< 0.01, 0.001 and no significant difference, respectively. Sample size n=3 in the figure.

In this study, the hydrogen peroxide content in different groups were determined. As shown in **Figure 4a**, no significant difference of hydrogen peroxide content was observed among the control group, HN11 treatment group, and AMF treatment group. However, the hydrogen peroxide content in leaf tissues of HN11+AMF treatment group diminished by 70.0%. While in root tissues, the hydrogen peroxide contents in HN11 treatment group, AMF treatment group, and HN11+AMF treatment group were significantly decreased when compared to the control group (**Figure 4a**). Elevated concentrations of reactive oxygen species were observed in the leaf and root tissues of the CK treatment group. The concentrations of reactive oxygen species following HN11 treatment, AMF treatment, and HN11+AMF treatment were markedly reduced compared to the CK group treatment (**Figure 4b**).

**Figure 5:**
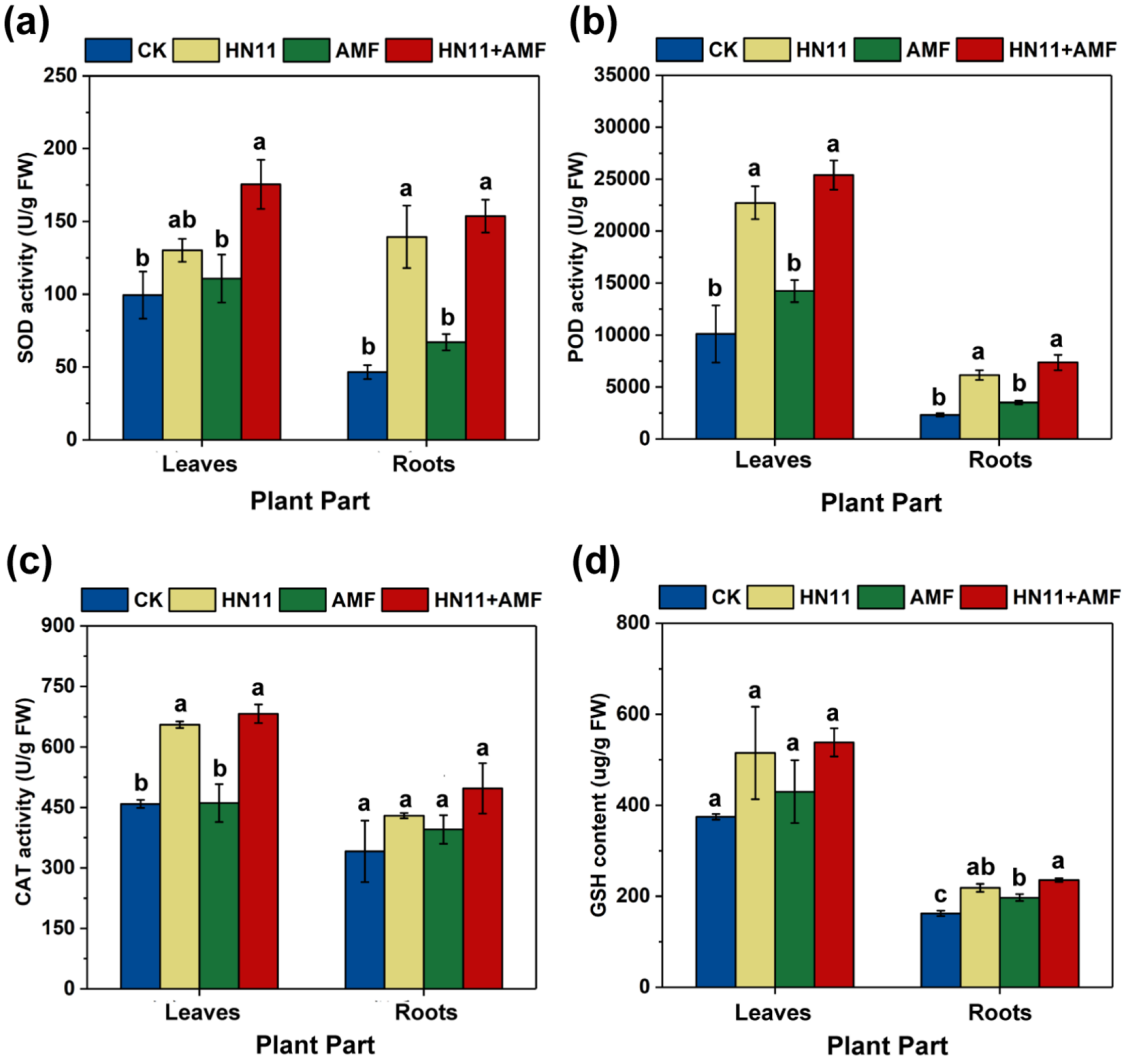
Antioxidant indexes of citrus plants after treatment with bacterial agents **(a)** Superoxide dismutase activity (SOD) **(b)** Peroxidase activity (POD) **(c)** Catalase activity (CAT) **(d)** Reduced glutathione content (GSH). CK is the control group, HN11 is the HN11 single inoculation treatment group, AMF is the AMF single inoculation treatment group, and HN11+AMF is the HN11+AMF double inoculation treatment group. The data in the figure are Mean ± SE. Different letters indicate significant differences between different treatments (*p*<0.05). The sample size in the figure is n=3.

In comparison to the control group, the SOD activity in the root system of citrus plants subjected to HN11+AMF exhibited a 230% rise (**Figure 5a**), POD activity rose by 216% (**Figure 5b**), CAT activity augmented by 48.8% (**Figure 5c**), and GSH content elevated by 45.1% (**Figure 5d**). The elevation of SOD and POD activities in leaves exhibited a pattern analogous to that in roots (**Figures 5a, b**). In addition, the significant increase of SOD, POD, and CAT activities in the group with HN11 was observed compared to the control group, while no significant difference was observed when compared to the group with HN11+AMF application. These results suggest that HN11 could plays a leading role in regulating the activity of antioxidant enzymes.

### 3.2 HN11 and AMF application inhibited the reproduction of *C*Las, promoted the plant growth, and altered soil microbial community under field conditions

#### 3.2.1 HN11 and AMF application inhibited the reproduction of *C*Las

**Figure 6:**
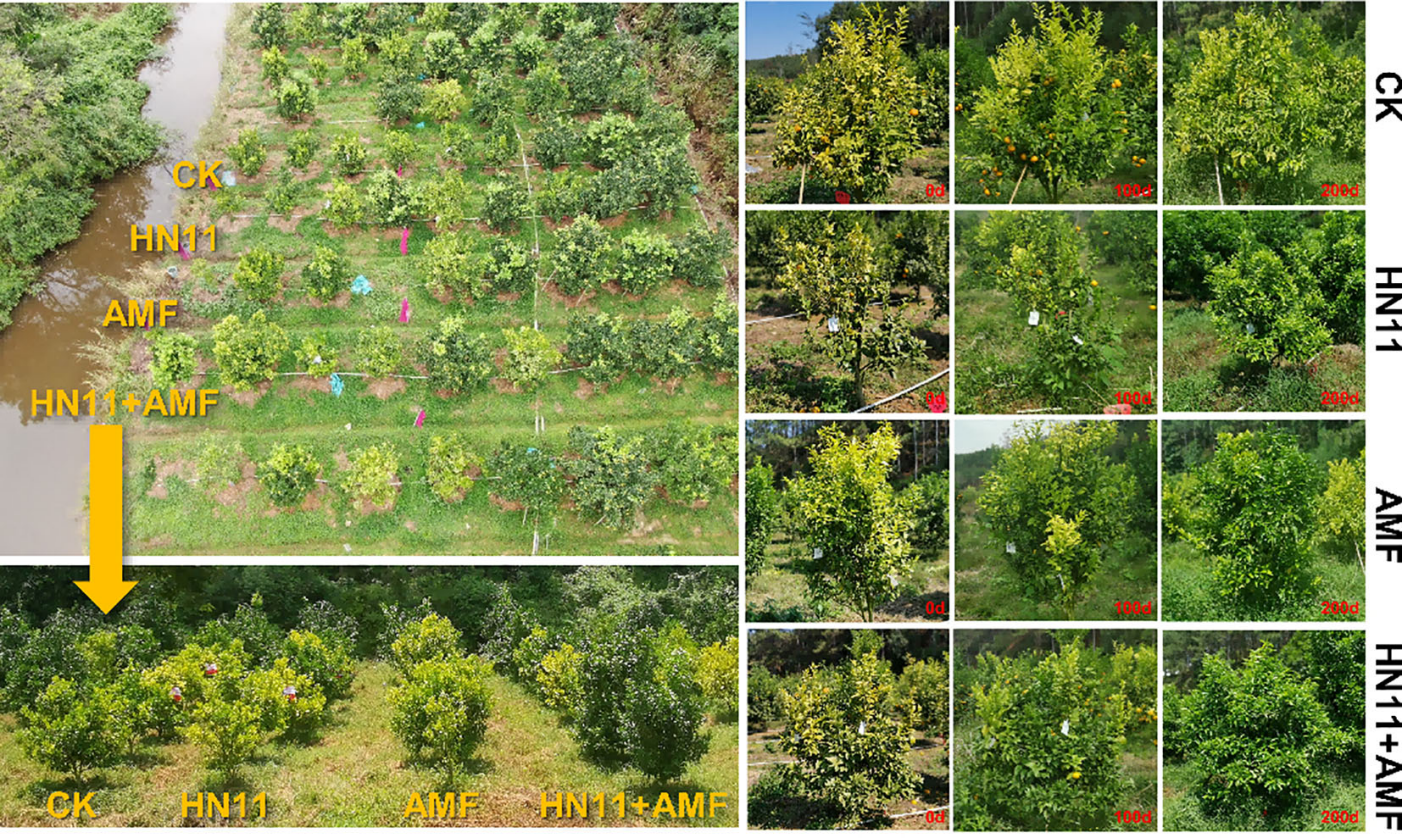
Citrus plant recovery status. CK is the control group, HN11 is the HN11 single inoculation treatment group, AMF is the AMF single inoculation treatment group, and HN11+AMF is the HN11+AMF double inoculation treatment group.

During 2021-2022, the effectiveness of HN11 treatment, AMF treatment, HN11 and AMF dual inoculation treatment against citrus HLB was confirmed in citrus orchards in Huizhou City, where the prevalence rate of HLB exceeded 80%. Citrus orchards impacted by HLB exhibited robust tender shoots and new growth following consistent treatment with HN11, AMF, and HN11+AMF for a duration of 7 months, respectively (**Figure 6**). Yellow and mottled leaves commenced reverting to green, and root activity progressively improved (**Figure 6**).

In February 2022, the concentration of *C*Las in citrus leaves treated with HN11+AMF diminished by 6.0×10^4^ copies/ng DNA, whereas the concentration of *C*Las in roots reduced by 2.0×10^5^ copies/ng DNA (**Table 4**). In April 2022, the concentration of HLB treated with HN11+AMF diminished by 2.0×10^4^ copies/ng DNA, whereas the concentration of *C*Las in root samples reduced by 6.0×10^5^ copies/ng DNA (**Table 4**). In June 2022, the *C*Las treated with HN11+AMF diminished by 2.0×10^4^ copies/ng DNA, whereas the *C*Las in the roots reduced by 4.0×10^4^ copies/ng DNA (**Table 4**). In addition, the decrease of *C*Las titers in the leaf and root samples after HN11 treatment, and AMF treatment was also observed. These results suggest that the three above treatments could inhibited the reproduction of *C*Las, and enhance the symptom progression of afflicted citrus fruits in the field.

**Table 4:** Changes in *C*Las titers (field experiment).

Tissue	Time	Parameter	CK	HN11	AMF	HN11+AMF
Leaves	December 2021	*C*Las	1.49×10^5^ ± 3.05×10^4^ **^a^**	9.84×10^4^ ± 2.74×10^4^ **^a^**	6.90×10^4^ ± 2.42×10^4^ **^a^**	1.09×10^5^ ± 3.77×10^4^ **^a^**
	February 2022	*C*Las	7.66×10^4^ ± 2.24×10^4^ **^a^**	9.17×10^3^ ± 1.32×10^3^ **^b^**	1.40×10^4^ ± 2.26×10^3^ **^b^**	9.72×10^3^ ± 2.68×10^3^ **^b^**
	April 2022	*C*Las	2.62×10^4^ ± 7.31×10^3^ **^a^**	1.89×10^3^ ± 4.05×10^2^ **^b^**	3.89×10^3^ ± 9.64×10^2^ **^b^**	3.23×10^3^ ± 1.14×10^3^ **^b^**
	June 2022	*C*Las	2.76×10^4^ ± 1.22×10^4^ **^a^**	4.99×10^2^ ± 8.85×10^1^ **^b^**	7.76×10^2^ ± 2.44×10^2^ **^b^**	1.18×10^3^ ± 8.22×10^2^ **^b^**
Roots	December 2021	*C*Las	3.00×10^5^ ± 1.22×10^5^ **^ab^**	1.03×10^6^ ± 3.31×10^5^ **^a^**	1.68×10^6^ ± 9.07×10^5^ **^a^**	1.51×10^5^ ± 8.02×10^4^ **^ab^**
	February 2022	*C*Las	2.42×10^5^ ± 1.10×10^5^ **^a^**	5.10×10^4^ ± 2.79×10^4^ **^a^**	8.14×10^4^ ± 5.08×10^4^ **^a^**	1.53×10^4^ ± 2.81×10^3^ **^a^**
	April 2022	*C*Las	6.25×10^5^ ± 5.80×10^5^ **^a^**	1.76×10^4^ ± 1.17×10^4^ **^b^**	1.46×10^4^ ± 8.07×10^3^ **^b^**	7.03×10^3^ ± 3.71×10^3^ **^b^**
	June 2022	*C*Las	5.75×10^4^ ± 2.02×10^4^ **^a^**	1.01×10^4^ ± 9.33×10^3^ **^b^**	1.02×10^4^ ± 4.98×10^3^ **^ab^**	3.09×10^3^ ± 2.88×10^3^ **^b^**

Different letters indicate differences between groups (p<0.05). The *C*Las unit is copies/ng DNA. The data presented in the table is Mean ± SE. The sample size in the figure is n=8. CK is the control group, HN11 is the HN11 single inoculation treatment group, AMF is the AMF single inoculation treatment group, and HN11+AMF is the HN11+AMF double inoculation treatment group.

#### 3.2.2 HN11 and AMF application enhanced the root growth of HLB-infected citrus trees

In comparison to the control group, the root activity of citrus subjected to HN11+AMF exhibited a 3.1-fold increase, the quantity of first-level new roots rose by 2.9-fold, the total root length augmented by 0.5-fold, the total root surface area expanded by 1.7-fold, and the number of root tips increased by 0.8-fold (**Table 5**). In addition, HN11 treatment also enhanced the root growth, with the increasing root activity, new roots, total root length, total root surface area, and root apex observed when compared to the control group. Furthermore, AMF treatment showed no significant impact on the root growth when compared to the control. These results demonstrate that the HN11 treatment and HN11+AMF treatment group exhibited notable enhancement in citrus root growth in the field.

**Table 5:** Root growth indicators of a field experiment.

Root growth indicators	CK	HN11	AMF	HN11+AMF
Root activity/μg g^-1^ h^-1^	188.28 ± 28.87 **^b^**	648.82 ± 54.29 **^a^**	311.74 ± 37.71 **^b^**	778.71 ± 54.38 **^a^**
New roots/strip	21.98 ± 3.93 **^c^**	67.27 ± 14.32 **^ab^**	45.60 ± 13.91 **^bc^**	84.82 ± 6.11 **^a^**
Total root length/cm	302.89 ± 11.83 **^c^**	447.98 ± 14.52 **^a^**	362.89 ± 15.90 **^b^**	452.97 ± 11.52 **^a^**
Total root surface area/cm^2^	57.54 ± 6.52 **^b^**	132.58 ± 8.53 **^a^**	79.60 ± 11.20 **^b^**	152.37 ± 15.68 **^a^**
Average root diameter/mm	1.42 ± 0.14 **^a^**	1.42 ± 0.11 **^a^**	1.25 ± 0.16 **^a^**	1.41 ± 0.14 **^a^**
Root tips/number	166.60 ± 9.29 **^a^**	232.00 ± 26.29 **^ab^**	198.80 ± 33.49 **^ab^**	261.20 ± 27.11 **^a^**
Root apex/number	168.60 ± 31.11 **^b^**	319.80 ± 51.16 **^a^**	227.40 ± 30.27 **^ab^**	304.00 ± 34.88 **^a^**
Root crossing/number	2.00 ± 0.84 **^a^**	2.80 ± 0.20 **^a^**	2.60 ± 0.60 **^a^**	3.20 ± 0.58 **^a^**

Different letters indicate differences between groups (*p*<0.05). n=5. CK is the control group, HN11 is the HN11 single inoculation treatment group, AMF is the AMF single inoculation treatment group, and HN11+AMF is the HN11+AMF double inoculation treatment group.

#### 3.2.3 The dual inoculation of HN11 and AMF alters the microbial community in field soil

##### 3.2.3.1 OTU annotation and analysis

To investigate the root microbial community of citrus before and after bacterial therapy, a microbiome investigation on the rhizosphere soil of CK1 group (diseased group), CK2 (healthy group), HN11 group, AMF group, and HN11+AMF group in a citrus orchard in Huizhou City was performed. All the OTU rarefaction curve’s transition to a smooth phase in the later phases signifies that the samples were adequate and appropriate (**Supplementary Figure 2**). Following clustering at a 100% similarity threshold, the mean quantities of bacterial OTUs recorded for the CK1 group (diseased group), CK2 (healthy group), HN11, AMF, and HN11+AMF samples were 1641, 1436, 1619, 1950, and 1599, respectively (**Supplementary Figure 3a**). The mean quantities of fungal OTUs recorded were 659, 619, 682, 668, and 721, respectively (**Supplementary Figure 3b**).

##### 3.2.3.2 HN11 and AMF application altered the diversity of bacteria and fungi in rhizosphere soil

The Shannon index and Chao1 index for bacteria and fungus in different groups were analyzed. As shown in **Supplementary Figures 4a, b**, no significant differences were seen in the Shannon index and Chao1 index values among the HN11, HN11+AMF therapy groups, and the CK1 (diseased group). Notable disparities were identified between the AMF treatment group and the CK1 (diseased group), with the AMF treatment group exhibiting markedly elevated Shannon index and Chao1 index values in comparison to the CK1 (diseased group). This suggests that the AMF-treated rhizosphere can attract a broader diversity of bacterial species in comparison to the CK1 (diseased group). In addition, the Shannon index and Chao1 index for fungus was also displayed (**Supplementary Figures 4c, d**). The Shannon index values for the CK1 (diseased group), AMF, and HN11+AMF treatment groups were markedly elevated compared to those of the CK2 (healthy group). This indicates that the rhizosphere fungal communities in the CK1 (diseased group), AMF, and HN11+AMF treatment groups exhibit greater diversity than those in the CK2 (healthy group). The heightened diversity may stem from the intrinsically rich and intricate microbial communities in sick plants or from the recruiting effects of AMF and HN11+AMF treatments.

**Figure 7:**
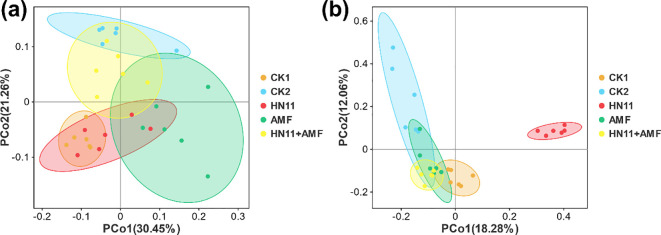
Principal Coordinates Analysis (PCoA) **(a)** Principal Coordinates Analysis for Bacteria **(b)** Principal Coordinates Analysis for Fungi. CK1 is the disease control group, CK2 is the healthy control group, HN11 is the HN11 single inoculation treatment group, AMF is the AMF single inoculation treatment group, and HN11+AMF is the HN11+AMF double inoculation treatment group.

In addition, the PCoA analysis of bacteria was performed (**Figure 7a**). The HN11+AMF treatment group closely resembles the CK2 (healthy group), with six sample replicates, signifying a substantial resemblance in microbial community structure between the two groups. The HN11+AMF group is substantially separated from the CK1 (diseased group), lacking sample replicates, which signifies a substantial disparity in species diversity. The PCoA analysis of fungus in different groups were also analyzed (**Figure 7b**). The HN11+AMF group closely resembles the CK2 (healthy group), with four sample replicates, demonstrating a significant match in microbial community structure between the two groups. The HN11+AMF group is substantially separated from the CK1 (diseased group), with no sample repeats, signifying a substantial disparity in species diversity. The findings indicate that following the HN11+AMF dual inoculation treatment, the species composition of the rhizosphere microbial community in afflicted citrus tends to shift towards a healthier condition. The results indicate that following the HN11+AMF dual inoculation therapy, the species makeup of the rhizosphere microbial community in sick citrus tends to shift towards a healthier state.

##### 3.2.3.3 HN11 and AMF application changed the microbial community composition and structure of rhizosphere soil

Among the five sample groups, the bacterial species were categorized into 24 phyla, 80 classes, 178 orders, 256 families, and 436 genera. Fungi comprised 10 phyla, 37 classes, 83 orders, 176 families, and 274 genera. The distribution of bacterial and fungal taxa at the phylum level was examined.

**Figure 8:**
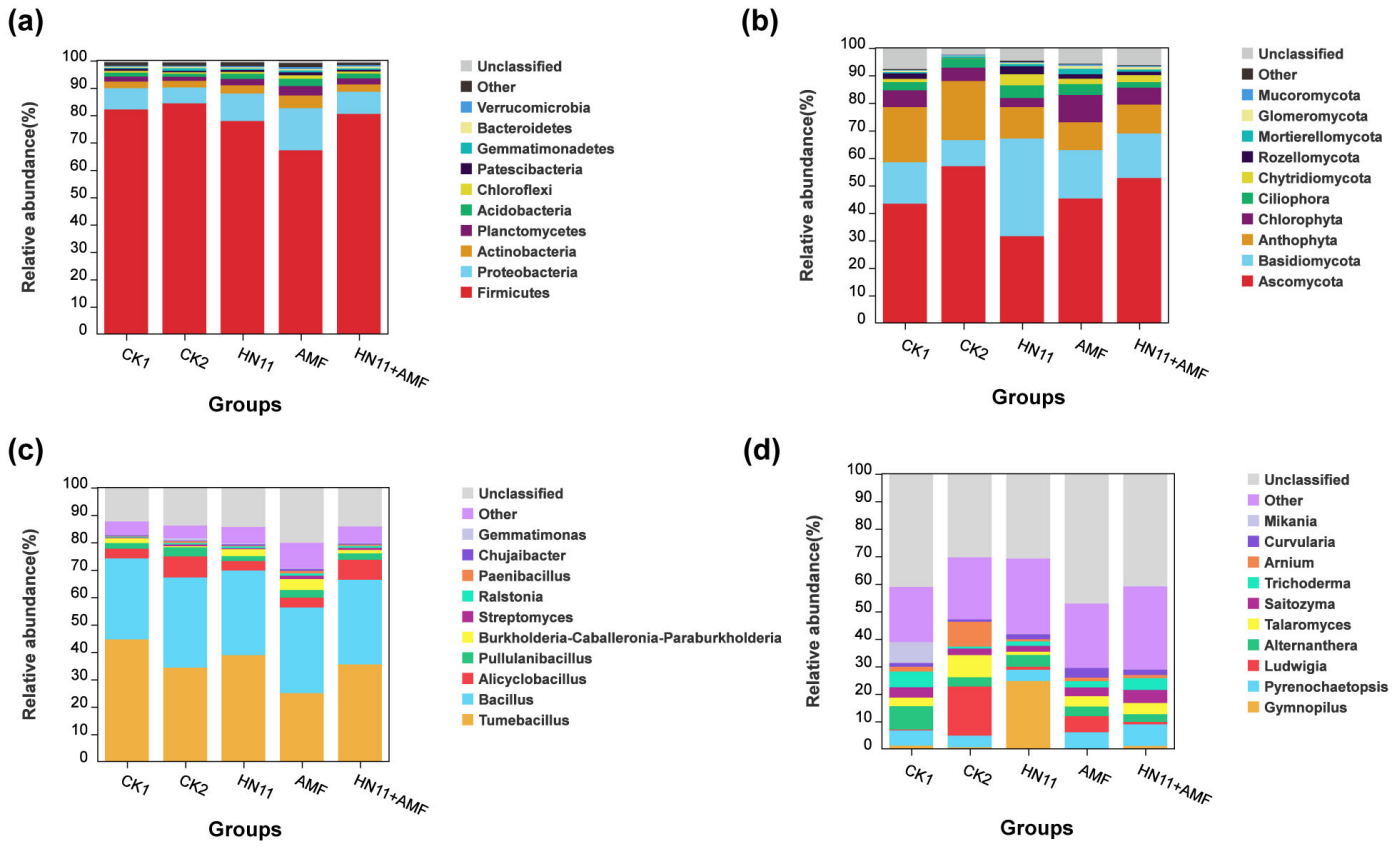
Species distribution at the phylum and genus levels **(a)** Species distribution of bacteria at the phylum level; **(b)** Species distribution of fungi at the phylum level; **(c)** Species distribution of bacteria at the genus level; **(d)** Species distribution of fungi at the genus level. CK1 is the disease control group, CK2 is the healthy control group, HN11 is the HN11 single inoculation treatment group, AMF is the AMF single inoculation treatment group, and HN11+AMF is the HN11+AMF double inoculation treatment group.

The distribution of bacterial species at the phylum level was analyzed. As shown in **Figure 8a**, the predominant bacterial phyla were Firmicutes and Proteobacteria, with Firmicutes as the most dominating group, comprising 67.14% to 84.28% of the relative abundance. The secondary major phylum was Proteobacteria, with a relative abundance between 5.79% and 15.37%. The proportions of Firmicutes in the CK2 (healthy group) and CK1 (diseased group) were 56.98% and 43.35%, respectively. Following treatment with the HN11+AMF group, the percentage of Firmicutes rose to 95.85%.

The distribution of fungal species at the phylum level was analyzed. As shown in **Figure 8b**, the predominant fungal phyla were Ascomycota and Basidiomycota, with Ascomycota representing the primary dominating group, comprising 31.53% to 56.98% of relative abundance. The secondary prevalent phylum was Basidiomycota, with a relative abundance between 9.42% and 35.49%.

The species distribution of the 10 most abundant bacteria and fungi at the genus level was enumerated. As shown in **Figure 8c**, the distribution of bacterial species at the genus level was analyzed. The relative abundance of *Bacillus* in the CK1 (diseased group) soil was 29.57%, lower than the healthy group (CK1) at 32.91%. Following treatment with HN11, AMF, and HN11+AMF, the relative abundance of Bacillus rose in comparison to CK1 (diseased group), attaining values of 30.90%, 31.25%, and 30.91% respectively, indicating a tendency towards enrichment akin to the healthy group (CK1). The relative abundance of Tumebacillus in the CK1 (diseased group) soil was 44.53%, surpassing that of the healthy group (CK1) at 34.22%. Following treatment with HN11, AMF, and HN11+AMF, the relative abundance of Tumebacillus diminished to 38.81%, 24.93%, and 35.42%, respectively, suggesting a shift towards the healthy cohort. Bacillus is a helpful microbe, and the rise in its relative abundance indicates that the inoculated HN11 strain can assist citrus in resisting HLB and restoring health. The distribution of fungal species at the genus level was also displayed (**Figure 8d**). Pyrenochaetopsis constituted the predominant group in the CK1 (diseased group), AMF, and HN11+AMF treatment groups, with a relative abundance ranging from 5.51% to 7.86%. The genus Gymnopilus constituted the predominant group in the HN11 treatment, with a relative abundance of 24.64%. Arnium constituted the predominant genus in the healthy cohort (CK1), exhibiting a relative abundance of 9.01%. This may result from the ecological environment’s impact on the structure and composition of the fungal microbial community, causing the formation of various dominating genera.

The combined inoculation of HN11 and AMF enhanced the rhizosphere microbial community structure of citrus afflicted by HLB, progressively directing the soil microbial population towards a more robust condition.

##### 3.2.3.4 HN11 and AMF application changed the microbial community functions of rhizosphere soil

The cluster heatmap analysis of the differential functions of rhizosphere bacterial populations subjected to various treatments was performed (**Supplementary Figure 5**). The findings demonstrate that the implementation of HN11 and AMF modified the functional profiles of the rhizosphere bacterial communities, which exhibited substantial differences from those of CK1 (diseased group) and CK2 (healthy group). In contrast to CK1 (diseased group), the upregulated differential functions in HN11, AMF, and HN11+AMF treatments were concentrated in the biosynthesis and metabolism of xenobiotics, polysaccharides, lipid metabolism, other secondary metabolites, terpenoids and polyketides, transport and catabolism, digestive system, signaling molecules and interactions, and environmental adaptation.

**Figure 9:**
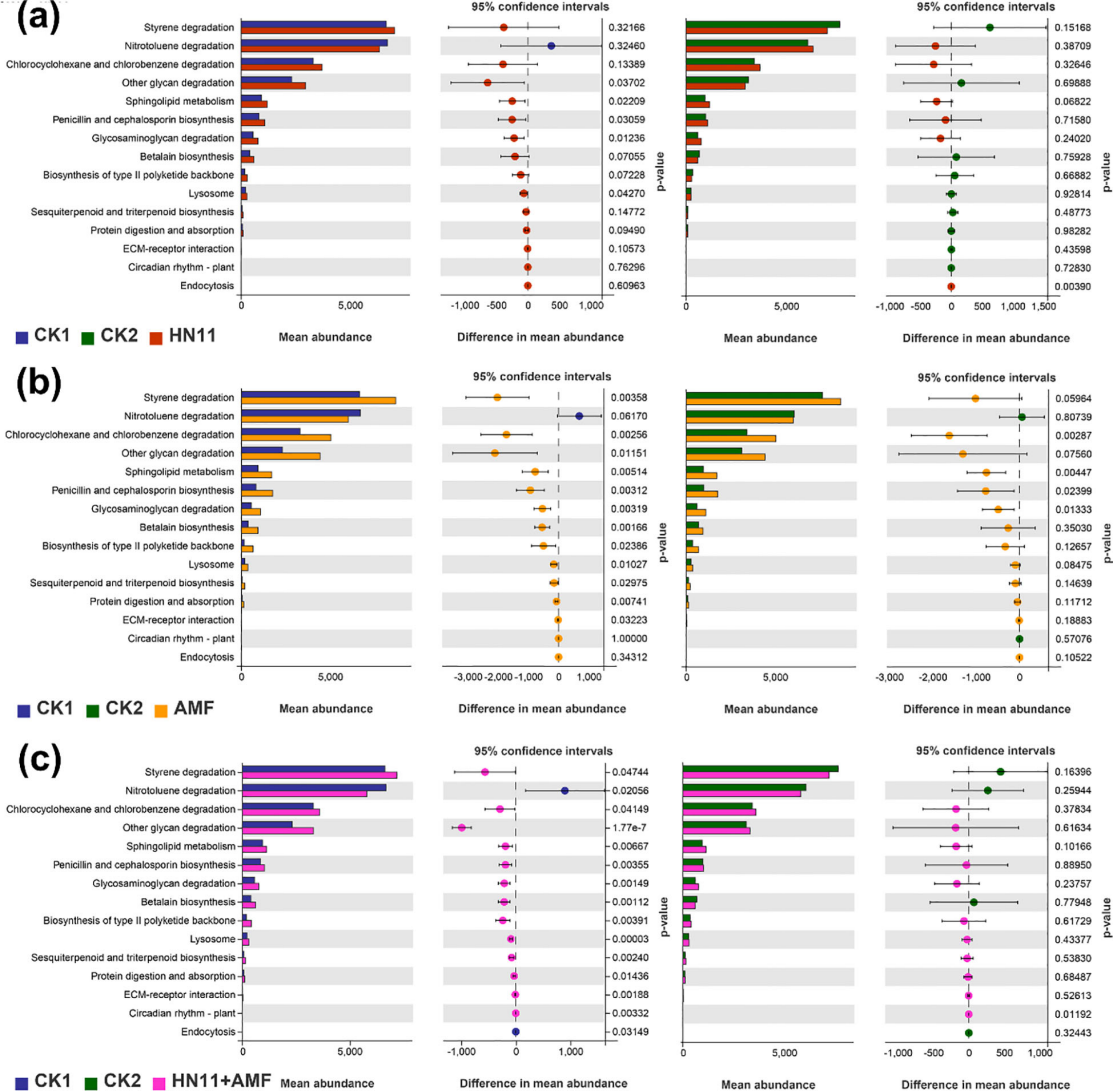
Relative abundance of key functional genes in rhizosphere bacterial communities predicted by PICRUSt 2. **(a)** Functional content of CK1, CK2, and the HN11 group; **(b)** Functional content of CK1, CK2, and the AMF group; **(c)** Functional content of CK1, CK2, and the HN11+AMF group. CK1 is the disease control group, CK2 is the healthy control group, HN11 is the HN11 single inoculation treatment group, AMF is the AMF single inoculation treatment group, and HN11+AMF is the HN11+AMF double inoculation treatment group.

The anticipated functional content analysis of CK1 (diseased group), CK2 (healthy group), and the HN11 group was analyzed (**Figure 9a**). In comparison to CK1 (diseased group), the HN11 treatment exhibited notable differences in the functional aspects of polysaccharide biosynthesis and metabolism (glycosaminoglycan degradation and degradation of other polysaccharides), lipid metabolism (sphingolipid metabolism), biosynthesis of additional secondary metabolites (biosynthesis of penicillin and cephalosporin), and transport and catabolism (lysosome). No notable variations were detected in any functional components. In comparison to CK2 (healthy group), the HN11 therapy group exhibited notable variations in transport and catabolism (endocytosis), whereas no significant abnormalities were observed in other functional components.

The anticipated functional content analysis of CK1 (diseased group), CK2 (healthy group), and the AMF group was also performed (**Figure 9b**). In comparison to CK1 (diseased group), the AMF treatment exhibited significant variations in the functional content of xenobiotic biosynthesis and metabolism (styrene degradation, chlorocyclohexane and chlorobenzene degradation), polysaccharide biosynthesis and metabolism (glycosaminoglycan degradation and degradation of other polysaccharides), lipid metabolism (sphingolipid metabolism), biosynthesis of other secondary metabolites (biosynthesis of penicillin and cephalosporin, betaine biosynthesis), terpenoid and polyketide metabolism (biosynthesis of type II polyketides, sesquiterpenoid and triterpenoid biosynthesis), transport and catabolism (lysosome), digestive system (protein digestion and absorption), and environmental adaptation (circadian rhythm in plants). No notable discrepancies were detected in the remaining functional components. In comparison to CK2 (healthy group), the AMF treatment group exhibited notable differences in the functional content related to xenobiotic biosynthesis and metabolism (degradation of chlorocyclohexane and chlorobenzene), lipid metabolism (sphingolipid metabolism), polysaccharide biosynthesis and metabolism (degradation of glycosaminoglycan), and the biosynthesis of various secondary metabolites (biosynthesis of penicillin and cephalosporin), while no significant differences were observed in other functional contents.

The anticipated functional content analysis of CK1 (diseased group), CK2 (healthy group), and the HN11+AMF group was also analyzed (**Figure 9c**). In comparison to CK1 (diseased group), the HN11+AMF treatment exhibited significant variations in the functional content of xenobiotic biosynthesis and metabolism (styrene degradation, nitrotoluene degradation, chlorocyclohexane and chlorobenzene degradation), polysaccharide biosynthesis and metabolism (glycosaminoglycan degradation and degradation of other polysaccharides), lipid metabolism (sphingolipid metabolism), biosynthesis of other secondary metabolites (biosynthesis of penicillin and cephalosporin, betaine biosynthesis), terpenoid and polyketide metabolism (biosynthesis of type II polyketides, sesquiterpenoid and triterpenoid biosynthesis), transport and catabolism (lysosome, endocytosis), digestive system (protein digestion and absorption), environmental adaptation (circadian rhythm in plants), and signal molecules and interactions (extracellular matrix-receptor interactions). In comparison to CK2 (healthy group), the HN11+AMF treatment group exhibited substantial variations in the functional content related to environmental adaptation (circadian rhythm in plants), whereas no notable alterations were observed in other functional contents.

The functional components of polysaccharide biosynthesis and metabolism (including glycosaminoglycan and other polysaccharide degradation), lipid metabolism (specifically sphingolipid metabolism), the biosynthesis of additional secondary metabolites (such as penicillin and cephalosporin), and transport and catabolism (lysosomal activity) were markedly more prevalent in the HN11, AMF, and HN11+AMF treatment groups in comparison to CK1 (the diseased group). Prior research has indicated that glycosaminoglycans and other polysaccharides function as possible immunometabolic regulators via several pathways, highlighting their involvement in modifying immunological responses elicited by HLB. Certain sphingolipids, owing to their antibacterial properties, may function as the primary defense against bacteria, indicating that sphingolipid metabolism could aid in the prevention of HLB. Moreover, other antibiotics, including penicillin and oxytetracycline, have demonstrated efficacy in eradicating or suppressing HLB infections, underscoring the importance of penicillin and cephalosporin biosynthesis in the treatment of HLB. Lysosomes can initiate cytotoxic autophagy, indicating their role in intracellular breakdown and recycling processes in HLB-affected oranges, facilitating the removal of intracellular debris and damaged organelles, thereby aiding in the restoration of plant health.

In summary, the HN11+AMF dual-inoculation therapy group demonstrated significant functional alterations due to the synergistic effects of the two microorganisms. There were several functional disparities in comparison to CK1 (diseased group), but fewer significant distinctions relative to CK2 (healthy group). This suggests that the microbial community’s functionality following HN11+AMF dual inoculation is advantageous for addressing citrus HLB, steering the afflicted citrus plants towards improved health.

**Figure 10:**
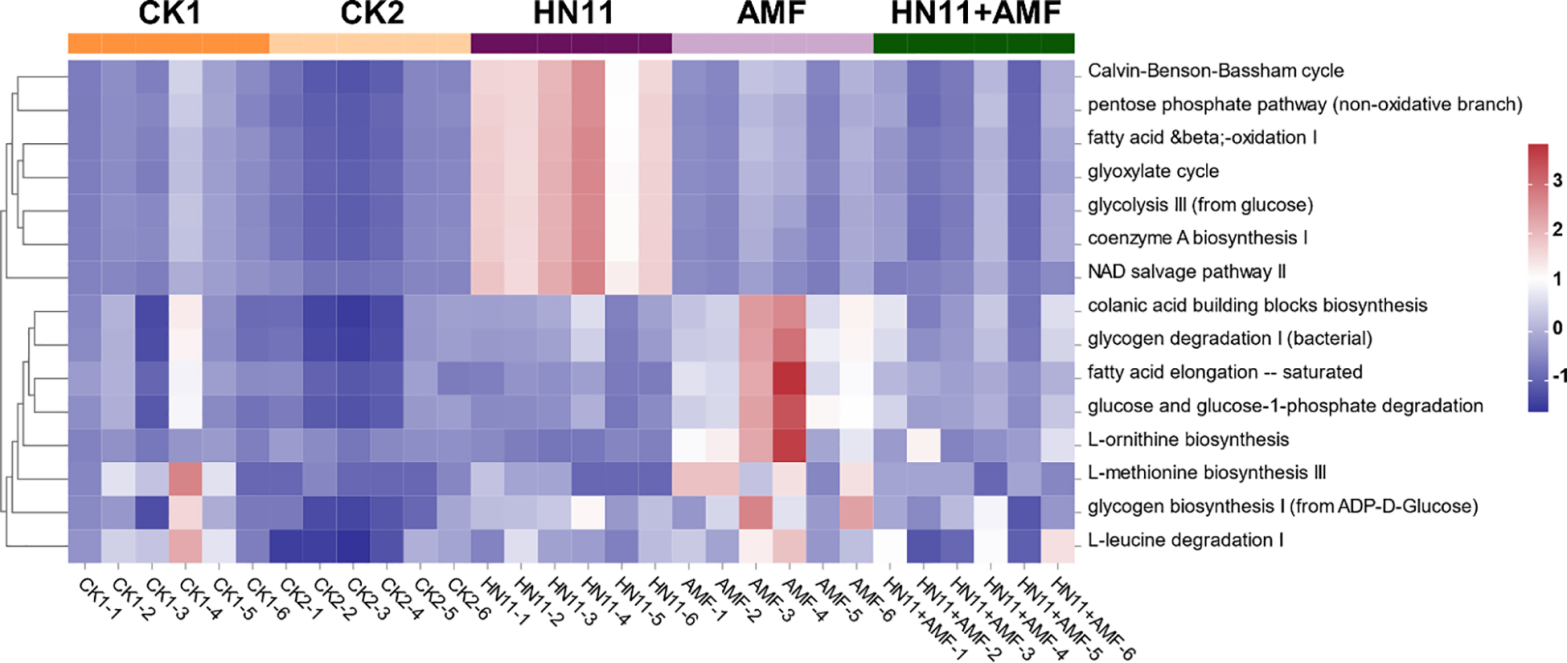
Cluster heatmap analysis of differential functions in rhizosphere fungal communities. CK1 is the disease control group, CK2 is the healthy control group, HN11 is the HN11 single inoculation treatment group, AMF is the AMF single inoculation treatment group, and HN11+AMF is the HN11+AMF double inoculation treatment group.

**Figure 10** displays the results of the cluster heatmap analysis of differential functions in rhizosphere fungal communities subjected to various treatments. The findings indicate that the implementation of HN11 and AMF modified the functional profiles of the rhizosphere fungal communities, which were markedly distinct from those of CK1 (diseased group) and CK2 (healthy group). The differential functionalities of HN11 relative to other treatment groups were predominantly concentrated in the C3 cycle, pentose phosphate route, fatty acid and β-oxidation pathway I, glyoxylate cycle, glycolysis pathway III, coenzyme A biosynthesis pathway I, and NAD salvage pathway II. The differential functions of AMF relative to other treatment groups were predominantly enriched in the chorismate biosynthesis pathway, glycogen degradation pathway I, saturated fatty acid elongation pathway, glucose and glucose-1-phosphate degradation pathway, L-ornithine biosynthesis pathway, L-methionine biosynthesis pathway III, ADP-D-glucose to glycogen pathway I, and L-leucine degradation pathway I. The functional disparities in the HN11+AMF dual-inoculation therapy were considerable as compared to the HN11 and AMF single-treatment groups, exhibiting a reduced number of enriched functional regions. This may result from the intricate interactions between HN11 and AMF in the soil.

## 4 Discussion

### 4.1 Control effect of dual inoculation of HN11 and AMF on citrus HLB

HLB is a bacterial disease affecting citrus, representing a significant danger to the citrus sector. This study examined the control effects and mechanisms of HN11 and AMF to identify feasible control techniques. These findings suggest that HN11 is a viable microbial control agent suitable for root application to manage citrus HLB. Comparable studies have been extensively documented. Nan et al. assessed the inhibitory impact and mechanism of *B. amyloliquefaciens* GJ1 against citrus HLB. The findings indicated that GJ1 significantly suppressed the proliferation of HLB pathogens and bolstered the resistance of citrus plants to the illness by stimulating plant defense mechanisms and elevating the activity of antioxidant enzymes (Nan et al., 2021). Munir et al. discovered that the indigenous citrus endophyte *B. subtilis* L1–21 have the capability to regulate HLB infections. *B. subtilis* L1–21 can suppress the proliferation and viability of HLB pathogens while augmenting the self-defense mechanisms of citrus by eliciting plant immune responses (Munir et al., 2022a). Asad et al. identified that the indigenous endophytic bacterium *B. subtilis* L1–21 can alleviate HLB infection by modulating nutrient metabolism in citrus. *B. subtilis* L1–21 can stimulate the production of endogenous plant growth hormones and antioxidants in citrus, facilitating root development and growth, consequently augmenting the immunity of citrus plants and diminishing pathogen invasion (Asad et al., 2021). This study is the first investigation into the efficacy of dual inoculation with *B. amyloliquefaciens* HN11 and AMF for the management of citrus HLB. HN11 and AMF significantly suppress the proliferation of HLB pathogens, foster plant growth via microbial colonization, mitigate disease progression, diminish plant immunological damage, stimulate antioxidant mechanisms, boost soil quality, and improve the absorption of nutrients. These works offer novel insights and methodologies for the microbiological management of citrus HLB.

Prior research has indicated that bacteria can proficiently manage plant diseases by inhabiting ecological niches. Some studies indicate that beneficial bacteria and AMF might interact at plant roots, cohabiting ecological niches and enhancing plant growth and disease resistance (Devi et al., 2022). Mendoza-Hernández et al. discovered that inoculation with AMF and probiotics can promote the growth of Mexican sweet orange and markedly decrease the prevalence of HLB. Furthermore, inoculation with AMF and probiotics markedly enhanced the bacterial density in the rhizosphere, especially beneficial bacteria that promote the health and disease resistance of Mexican sweet orange (Mendoza-Hernández et al., 2019). Soil nutrients can affect the microbial populations on leaf surfaces and in the rhizosphere, consequently diminishing the occurrence of HLB. This suggests that the interaction between soil and plant internal bacteria is vital for combating HLB (Zhou et al., 2021). Consequently, these reports correspond with the conclusions of this study. HN11 could decrease the concentration of HLB pathogens in the roots of infected citrus by occupying the ecological niches of these pathogens, with HN11–10^8^ demonstrating the most pronounced effect, dramatically lowering the levels of HLB pathogens. HN11 may additionally infiltrate citrus root tissues via lesions induced by AMF infection or at the junctions of primary roots and mycorrhizae. Consequently, when a specific quantity of advantageous bacteria HN11 occupies these essential ecological niches in advance, it can inhibit subsequent infections by HLB pathogens. Despite the limited population of HN11 infiltrating the roots, prolonged and consistent administration can adapt HN11 to the root environment, facilitating its proliferation. The presence of HN11 and AMF may also attract other beneficial bacteria to colonize the roots, collectively occupying vital ecological niches and inhibiting further infection by HLB pathogens.

### 4.2 Effects of dual inoculation of HN11 and AMF on citrus growth

HN11 and AMF are two advantageous bacteria for plants that can mitigate plant diseases via distinct ways. HN11 is a soil-dwelling plant growth-promoting strain that can alleviate illnesses by augmenting nutrient absorption and bolstering plant disease resistance (Zhang et al., 2016). Conversely, AMF is a soil fungus that establishes a symbiotic association with plant roots, referred to as mycorrhizae, which enhances the plant’s capacity to absorb nutrients and water, hence augmenting its resistance to disease (Bennett and Groten, 2022). This study’s findings demonstrate that dual inoculation with HN11 and AMF markedly enhances the growth characteristics of citrus afflicted by HLB, including plant height, root length, plant biomass, root-to-shoot ratio, leaf area, and chlorophyll content. These data corroborate the efficacy of dual inoculation as a therapeutic approach for HLB-infected citrus. The study indicates that dual inoculation treatment significantly enhances root growth, implying that future field studies should prioritize the enhancement of root development.

Research indicates that AMF can form a symbiotic association with plant roots, thereby improving nutrient uptake and disease resistance (Begum et al., 2019). HN11 is a potent biocontrol agent capable of suppressing pathogen proliferation while simultaneously enhancing plant development (Lin et al., 2017). Consequently, simultaneous inoculation with these two treatments can exploit their synergistic effects to enhance plant growth and disease resistance. While the outcomes of the pot experiment including citrus align closely with expectations, such trials may not entirely represent field situations. Consequently, additional field trials are required to confirm the viability and efficacy of this dual inoculation treatment. Moreover, additional factors affecting growth metrics, like climate and soil conditions, must be considered.

HLB has been observed to impair citrus roots by suppressing root activity and decreasing the formation of new roots (Atta et al., 2020). A study on citrus rhizosphere microorganisms revealed that inoculation with microbial agents enhances chlorophyll content and photosynthetic efficiency, thus enhancing citrus growth and yield (Castellano-Hinojosa et al., 2021). This study’s results indicate that inoculation with HN11 and AMF successfully mitigates root rot, promotes root development, and bolsters plant immunity and disease resistance. Consequently, dual inoculation may assist in reducing root necrosis induced by HLB infection, thereby lessening the advancement of HLB symptoms in citrus.

### 4.3 Effects of dual inoculation of HN11 and AMF on citrus immune response, antioxidant capacity, and soil health

Microorganisms in the rhizosphere can modulate plant immune responses in multiple ways, thereby diminishing the effects of diseases on plants. Moreover, rhizosphere microorganisms can influence the plant’s antioxidant system, thereby augmenting its resistance to pathogens (Li et al., 2021). Research indicates that citrus HLB is an immune-related affliction induced by pathogens, with its manifestation linked to the dysregulation of the plant immune system. Concurrently, HLB induces the generation of substantial quantities of ROS, including hydrogen peroxide, within the plant, exacerbating the impairment of the plant’s immune system and cellular architecture (Ma et al., 2022). This study examined the impact of HN11 and AMF treatments on the immune response and cellular apoptosis in citrus plants affected by HLB infection. The findings indicated that HN11 and AMF treatments markedly diminished hydrogen peroxide and reactive oxygen species levels in HLB-infected citrus plants, concurrently relieving HLB symptoms. The combination inoculation of HN11 and AMF may considerably enhance the growth characteristics of HLB-infected citrus, consequently decreasing hydrogen peroxide levels through growth restoration, alleviating pathogen infection, and safeguarding plant cells from damage.

Callose deposition is crucial in plant immunological responses. Starch metabolism is associated with callose deposition, as starch is hydrolyzed into glucose to supply energy for callose deposition during pathogen assault in plants (Wang et al., 2021). This investigation revealed that, in contrast to leaf tissues, callose deposition and starch concentration diminished more markedly in root tissues following microbial treatment. In contrast to current results about microbial-induced callose deposition for disease resistance, this may be attributed to the fact that roots are among the most common sites of interaction between plants and soil microbes. Certain rhizosphere bacteria synthesize hydrolases that decompose callose into monosaccharides, hence enhancing nutrient absorption and use, and affecting plant growth and development.

SOD, POD, and CAT are the primary antioxidant enzymes in plants, proficient in scavenging reactive oxygen species and safeguarding cells from oxidative harm. GSH is a significant non-enzymatic antioxidant with reducing capability, capable of reacting with oxidative agents and stabilizing their structure (Batool et al., 2020). This study examined the activity of antioxidant enzymes and the levels of non-enzymatic glutathione in plants subjected to various microbial treatments. The findings indicated that the levels of SOD, POD, and GSH in plants only treated with HN11, and treated with HN11 and AMF were significantly elevated, demonstrating that HN11 could effectively augment the antioxidant capacity of plants and mitigate oxidative damage induced by HLB infections. The results indicated variations in the activity of several antioxidant enzymes across distinct plant tissues. SOD and GSH primarily facilitate antioxidant action in roots, CAT mostly enhances antioxidant activity in leaves, whereas POD functions in both leaves and roots. This indicates that various antioxidant enzymes have specific functions in different plant regions, constituting a cohesive antioxidant defense system. Consequently, while assessing the antioxidant capacity of plants, it is essential to include the performance across several components to thoroughly evaluate their overall antioxidant potential.

Soil enzyme activity significantly affects plant growth and development by playing a vital role in essential processes including nutrient cycling and organic matter decomposition in the soil (Ren et al., 2021). This research assessed the impact of several microbial treatments on soil enzyme activity via pot experiments. The synergistic application of HN11 and AMF may effectively enhance the growth of HLB-infected citrus and rehabilitate soil health.

## Data Availability

The datasets presented in this study can be found in online repositories. The names of the repository/repositories and accession number(s) can be found below: https://www.ncbi.nlm.nih.gov/, PRJNA997928.
