# Supplementary material for: The application of Bacillus amyloliquefaciens and Arbuscular Mycorrhizal Fungi displays curative effects on Citrus Huanglongbing
Source: Front Plant Sci. 2025 Aug 29;16:1636064. doi: 10.3389/fpls.2025.1636064 (PMC12427124; doi:10.3389/fpls.2025.1636064)
Supplement: Supplementary file 1 [file DataSheet1.docx]

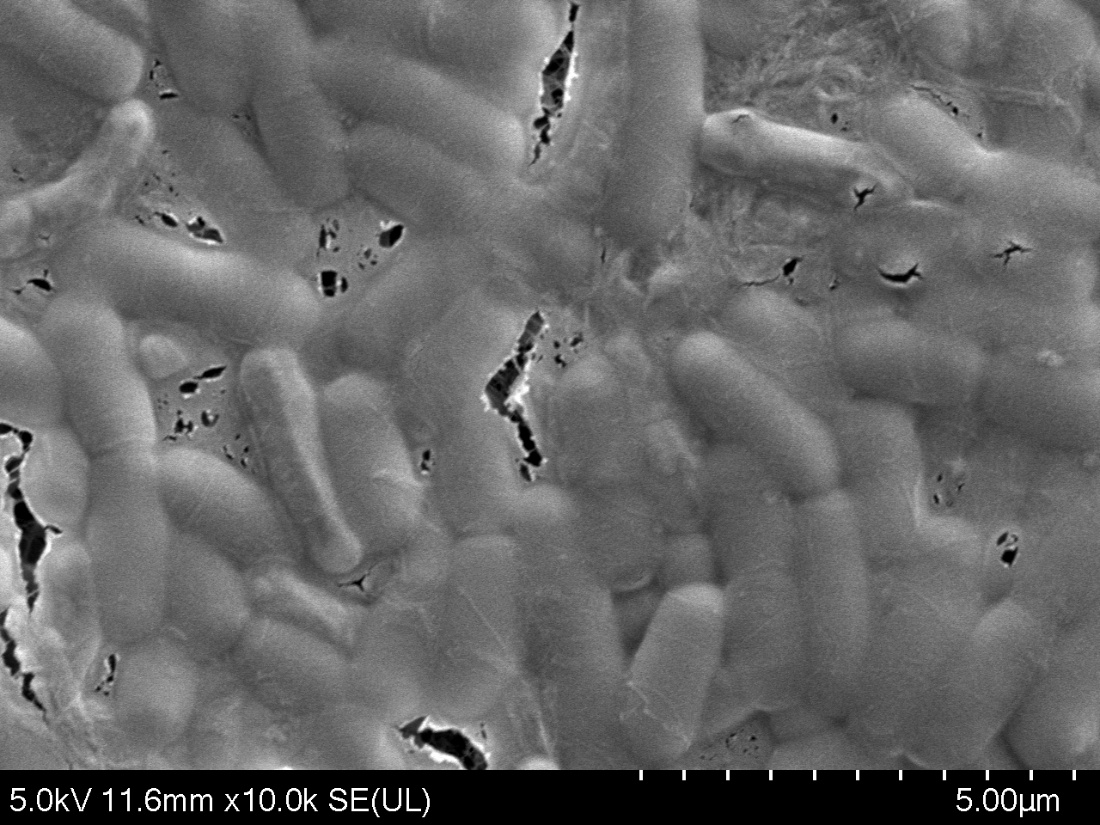


**Supplemental Figure 1:** Morphological characteristics of HN11 under scanning electron microscope


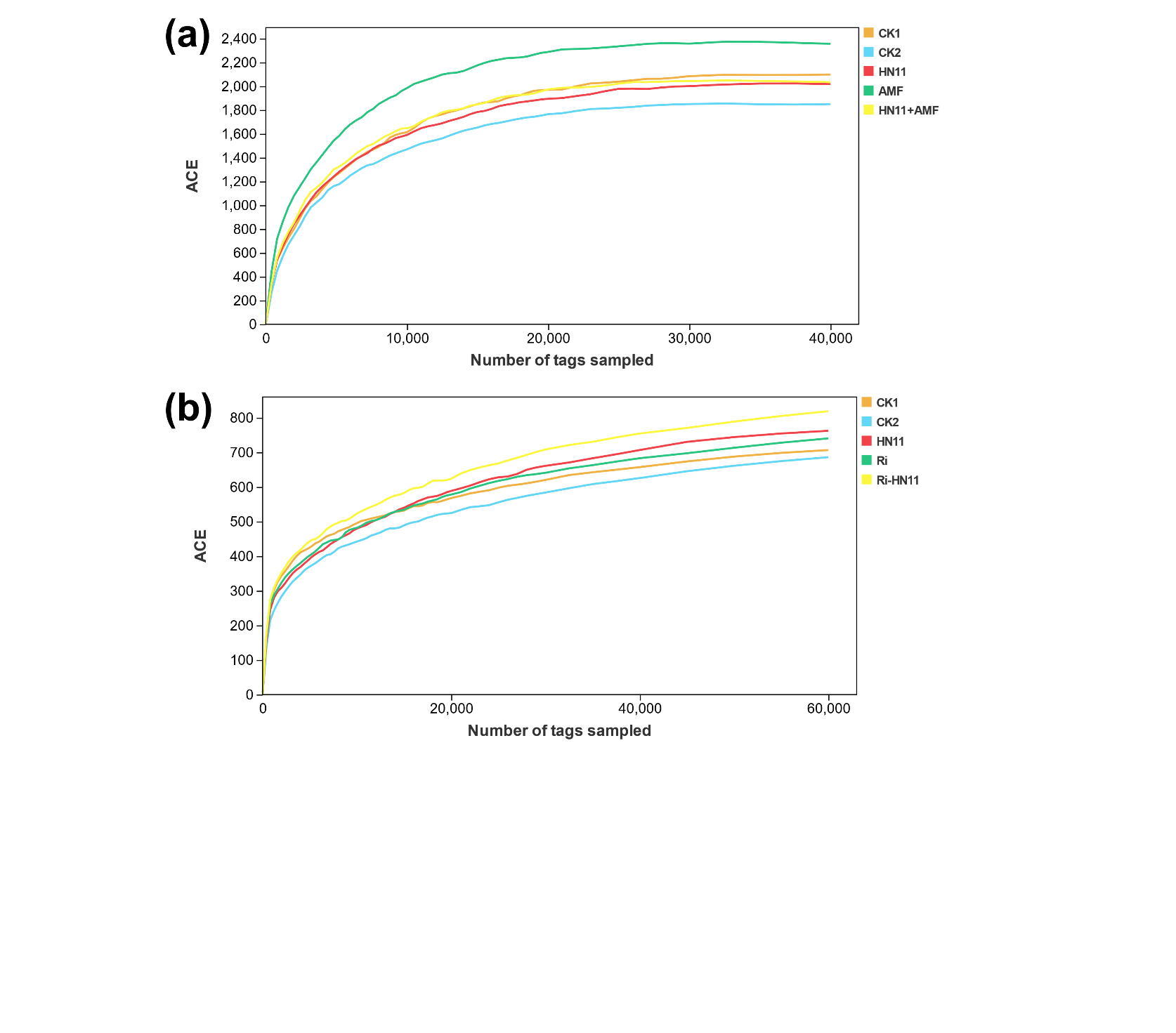


**Supplemental Figure 2.** Sample Dilution Curves. (a) Bacterial Sample Dilution Curve. (b) Fungal Sample Dilution Curve. CK1 is the disease control group, CK2 is the healthy control group, HN11 is the HN11 single inoculation treatment group, AMF is the AMF single inoculation treatment group, and HN11+AMF is the HN11+AMF double inoculation treatment group.


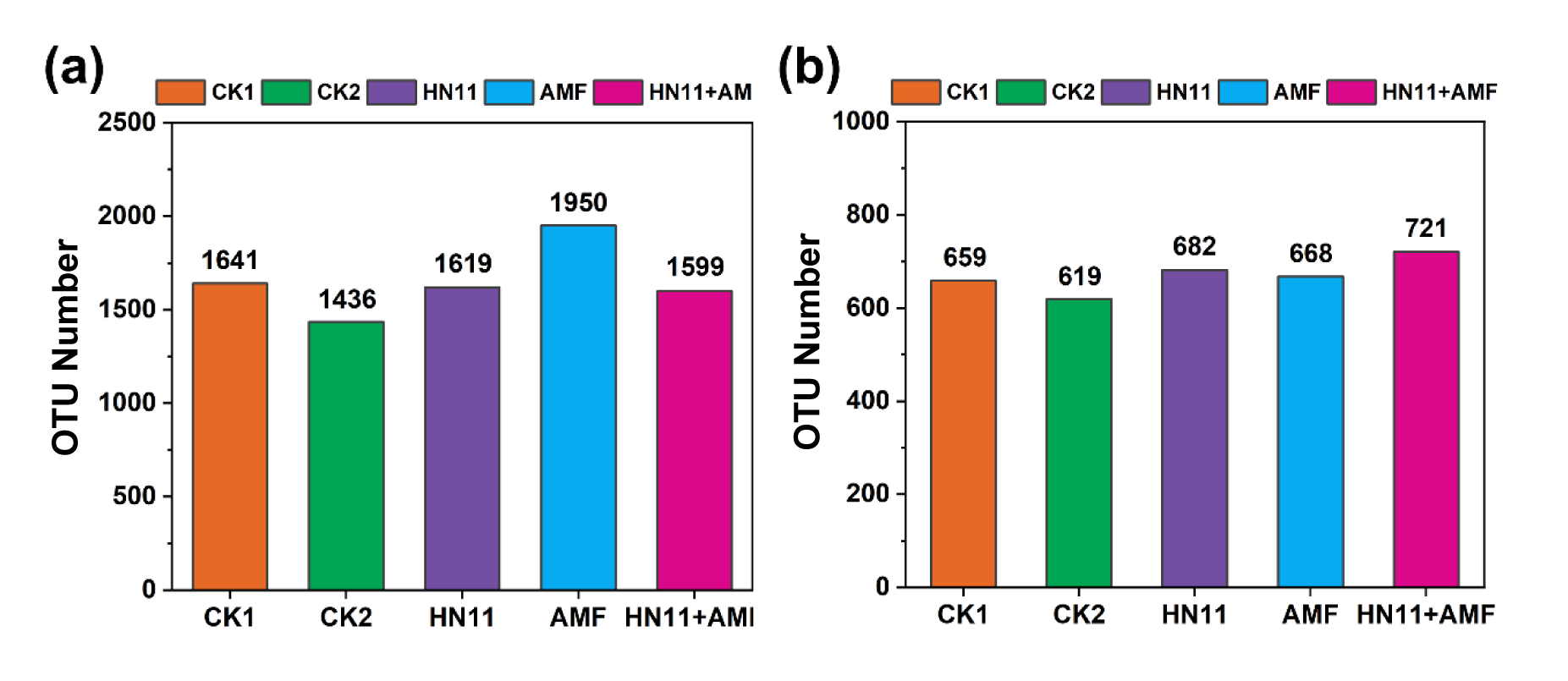


**Supplemental Figure 3.** Average OTU number distribution diagram (a) Average bacterial OTU number distribution diagram (b) Average fungal OTU number distribution diagram. CK1 is the disease control group, CK2 is the healthy control group, HN11 is the HN11 single inoculation treatment group, AMF is the AMF single inoculation treatment group, and HN11+AMF is the HN11+AMF double inoculation treatment group.


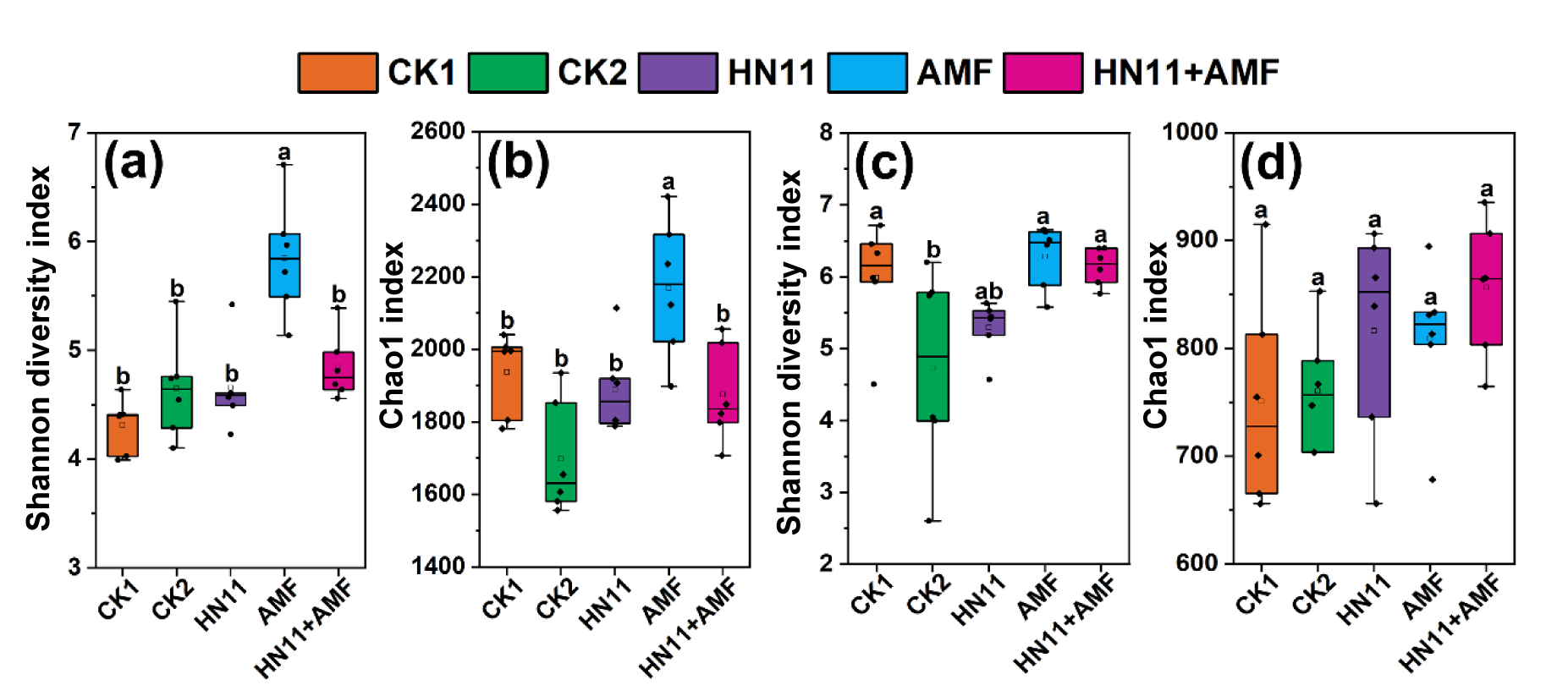


**Supplemental Figure 4.** Shannon Index and Chao1 Index. (a) Shannon index for bacteria. (b) Chao1 index for bacteria. (c) Shannon index for fungi. (d) Chao1 index for fungi. CK1 is the disease control group, CK2 is the healthy control group, HN11 is the HN11 single inoculation treatment group, AMF is the AMF single inoculation treatment group, and HN11+AMF is the HN11+AMF double inoculation treatment group. The data in the figure are presented as Mean ± SE. Statistical analysis was performed using One-way ANOVA, and different letters indicate significant differences between treatments (P < 0.05, DMRT method).


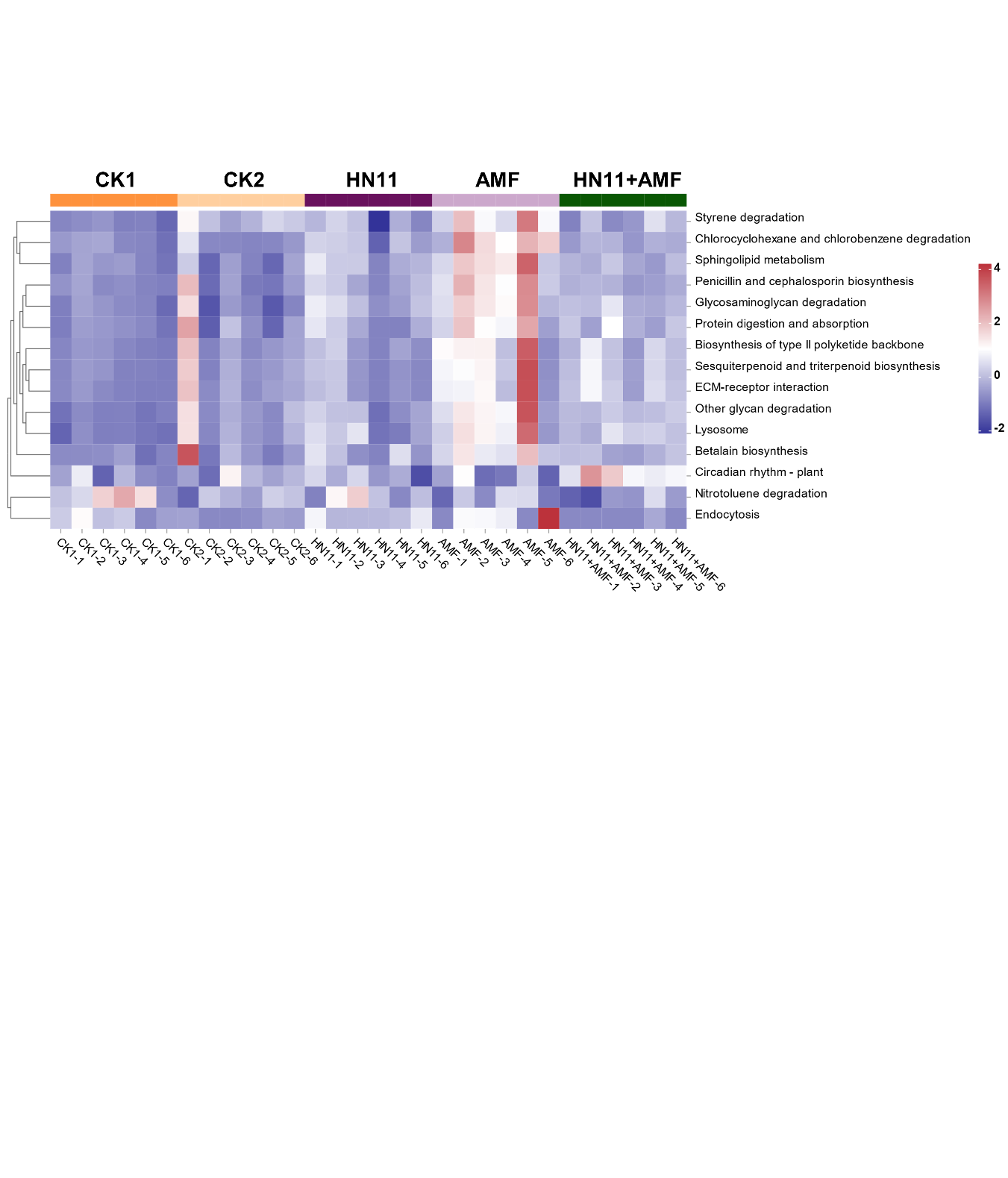


**Supplemental Figure 5.** Cluster Heatmap Analysis of Differential Functions in Rhizosphere Bacterial Communities. CK1 is the disease control group, CK2 is the healthy control group, HN11 is the HN11 single inoculation treatment group, AMF is the AMF single inoculation treatment group, and HN11+AMF is the HN11+AMF double inoculation treatment group.
